# Fully Sensitized Upconversion Nanoparticles as Efficient Catalysts for NIR‐Driven UV Photochemistry

**DOI:** 10.1002/anie.202511247

**Published:** 2025-09-16

**Authors:** Naomi Weitzel, Armaz Tsutskiridze, Julia Bramowski, Burkhard König, Thomas Hirsch

**Affiliations:** ^1^ Institute of Analytical Chemistry Chemo‐ and Biosensors University of Regensburg Universitätsstraße 31 93040 Regensburg Germany; ^2^ Institute of Organic Chemistry University of Regensburg Universitätsstraße 31 93040 Regensburg Germany

**Keywords:** Cycloaddition, Doping, Luminescence, Nanoparticles, Photocatalysis

## Abstract

Biological photosynthesis harnesses energy from multiple photons to drive complex chemical transformations. In contrast, chemical photocatalysis typically relies on single‐photon excitation, limiting its applicability in high‐energy‐demanding reactions. Upconversion nanoparticles (UCNPs), which can convert multiple low‐energy near‐infrared (NIR) photons into a single higher‐energy photon, offer a promising solution. We synthesized and systematically improved NaYbF_4_:Tm@NaYF_4_ nanoparticles, focusing on sensitizer concentration, dopant spacing, and shell thickness to enhance ultraviolet (UV) and blue emission. Compared to low doped NaYF_4_:Yb, Tm systems, our nanoparticles exhibited significantly improved brightness, with a 210‐fold enhancement in UV emission at 345 nm. Using these UCNPs as heterogeneous photocatalysts, we achieved efficient [2 + 2] photocycloadditions and Paternò–Büchi reactions under 980 nm excitation, with turnover numbers (TON) exceeding 290,000 and turnover frequencies (TOF) up to 8.52 s^−1^. Additionally, the UCNP catalysts were readily recoverable. Our results provide a rational framework for tailoring UCNPs for energy‐demanding photochemical reactions and establish their potential in synthetic and biomedical applications that require deep‐tissue, low‐phototoxicity excitation.

## Graphical abstract

Engineered NaYbF_4_:Tm^3+^@NaYF_4_ nanoparticles, featuring a fully sensitized Yb^3+^ lattice and an optimized core–shell architecture, convert five 980 nm photons by sequential absorption into a single 345 nm ultraviolet (UV) photon. This unprecedented anti‐Stokes shift enables energy‐demanding [2 + 2] photocycloadditions under mild, near‐infrared (NIR) excitation—unlocking high‐energy photochemistry with exceptional efficiency.

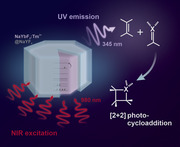

## Introduction

**Scheme 1:**
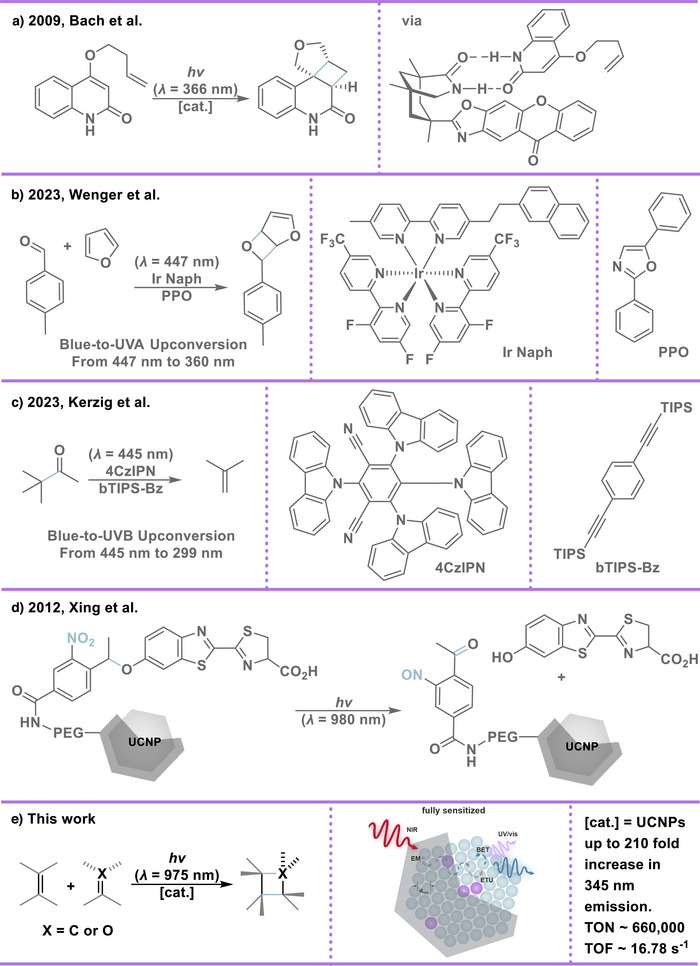
Synthetic examples of [2 + 2] photocycloadditions. a) UVA induced enantioselective intermolecular [2 + 2] photocycloadditions of isoquinolone mediated by a chiral hydrogen‐bonding template. b) Organometallic bichromophore – Ir Naph and annihilator – PPO – driven UVA photoreactions via blue‐to‐UV triplet–triplet annihilation (TTA) upconversion. Ir Naph: naphthalene‐tethered iridium(III) complex, PPO: diphenyloxazole. c) organic dye 4CzIPN and annihilator bTIPS‐Bz‐driven UVB‐mediated Norrish type I cleavage via blue‐to‐UV TTA upconversion. bTIPS‐Bz: triisopropylsilyl‐benzene, 4CzIPN: 1,2,3,5‐Tetrakis(carbazol‐9‐yl)‐4,6‐dicyanobenzene. d) NIR‐sensitized UVA‐mediated uncaging of covalently bound D‐luciferin on surface‐modified UCNPs. PEG: polyethylene glycol. e) UCNPs as catalysts for NIR‐driven UV [2 + 2] mediated photocycloadditions. EM: energy migration, BET: back‐ernergy transfer, ETU: energy transfer upconversion.

Over the past two decades, photoinduced synthetic and catalytic processes – where light serves as a traceless reagent – have gained significant attention, unlocking a variety of reactions that enable novel synthetic methodologies.^[^ ^1^, ^2^, ^3^, ^4^, ^5^, ^6^, ^7^, ^8^ ^]^ Among these, the [2 + 2] photocycloaddition stands out as a classical and widely applied photochemical reaction. However, typically one of the two olefins must be excited by highly energetic photons in the ultraviolet (UV) region of the electromagnetic spectrum.^[^ ^9^ ^]^ Significant progress has been made in the design and development of photocatalytic systems that use twofold activation for the selective construction of cyclobutenes by modulating the triplet reactivity of olefins. This approach typically involves the bathochromic shift of the absorption spectrum of conjugated olefins with specific functionalities, achieved through hydrogen bonding^[^ ^10^, ^11^, ^12^ ^]^ or Brønsted/Lewis acid catalysis,^[^ ^13^, ^14^, ^15^, ^16^ ^]^ followed by triplet sensitization^[^ ^3^ ^]^ via an external photocatalyst. In 2009, Bach et al.^[^ ^17^ ^]^ demonstrated that a chiral template‐based catalyst enables enantio‐ and diastereoselective intramolecular [2 + 2] UVA‐promoted photocycloadditions of quinolones, where the supramolecular complex formed between the chiral template and substrate selectively shields one face, promoting high enantiofacial differentiation (Scheme 1a). Yoon et al.^[^ ^18^ ^]^ achieved high reactivity and stereocontrol in cyclobutane synthesis through a tandem photoredox and chiral Lewis acid catalysis process.

Ultraviolet light (*λ* < 350 nm) is scarce in the solar spectrum, and available light sources, such as mercury lamps^[^ ^19^ ^]^ or xenon arc lamps,^[^ ^20^ ^]^ suffer from short lifespans and low energy conversion efficiencies. While UV light emitting diodes (LEDs) offer a more sustainable alternative and have seen improvements in power output, their application in high‐energy photochemistry is often limited by poor optical penetration in biological matrices and/or colored solutions, surface‐restricted excitation geometries, and the need for special laboratory glassware. These challenges have led to growing interest in multiphoton upconversion systems. For example, triplet‐triplet annihilation upconversion (TTA‐UC)^[^ ^21^, ^22^ ^]^ can efficiently convert visible light into UV light in situ, enabling high‐energy‐demanding photochemical transformations to proceed under milder, more accessible conditions. In 2023, Wenger et al.^[^ ^23^ ^]^ introduced a bichromophore photosensitizer comprising a naphthalene‐tethered iridium(III) complex (Ir‐Naph) and the annihilator diphenyloxazole (PPO), which, in tandem, enhanced blue‐to‐UV upconversion efficiency and enabled UVA‐driven reactions, including the Paternò–Büchi [2 + 2] cycloaddition (Scheme 1b). In the same year, Kerzig et al.^[^ ^24^ ^]^ reported an double substituted triisopropylsilyl annihilator (bTIPS‐Bz) with an emission maximum in the UVB region (*λ* = 299 nm), enabling blue‐to‐UVB upconversion when combined with a carbazole dicyanobenze‐based organic sensitizer (4Cz‐IPN). Through Förster resonance energy transfer (FRET), singlet‐excited bTIPS‐Bz activates and promotes Norrish type I cleavage of aliphatic carbonyl compounds (Scheme 1c).

Despite significant advances in generating and applying UV light via upconversion for photochemical transformations, most studies have focused on higher‐energy visible light, particularly blue‐to‐UV conversion. However, such light is strongly absorbed and scattered in biological tissues and colored media,^[^ ^25^ ^]^ significantly limiting its penetration depth for photochemical and theranostic applications.^[^ ^26^ ^]^ In the context of photodynamic therapy, these limitations are so pronounced that they have been referred to as its “Achilles' heel”.^[^ ^27^ ^]^ Additionally, its high energy increases the risk of photodamage^[^ ^26^, ^28^, ^29^ ^]^ and unwanted side reactions.^[^ ^30^ ^]^ Consequently, the demand for red and especially near‐infrared (NIR) driven photochemistry increased^[^ ^31^ ^]^ as NIR light penetrates more deeply with less scattering and lower phototoxicity, making it more suitable for biomedical^[^ ^32^, ^33^, ^34^ ^]^ and complex photochemical applications.^[^ ^32^, ^35^, ^36^, ^37^, ^38^ ^]^

Although NIR‐to‐visible upconversion‐induced photocatalysis has experienced significant advances, to the best of our knowledge, no photocatalytic systems have been explicitly designed for NIR‐driven UV photochemistry.^[^ ^21^, ^39^, ^40^ ^]^ Commonly employed upconversion strategies, such as TTA‐UC,^[^ ^21^, ^22^ ^]^ second harmonic generation (SHG),^[^ ^41^ ^]^ and two‐photon absorption (TPA),^[^ ^42^, ^43^ ^]^ function by combining the energy of two photons to generate a single photon of higher energy. However, the energy of two NIR photons (*λ* ≥ 800 nm, E ≤ 1.5498 eV) remains insufficient to produce a photon in the UV range (*λ* ≤ 380 nm, E ≥ 3.2627 eV). This fundamental limitation renders the abovementioned conventional upconversion methods unsuitable for NIR‐to‐UV conversion. To address this challenge, we identified upconversion nanoparticles (UCNPs)^[^ ^44^ ^]^ as a promising alternative. Unlike other strategies, UCNPs are capable of sequentially absorbing multiple low‐energy photons, often more than two, and emitting a single photon with sufficient energy to reach the UV range.

In more detail, UCNPs are lanthanide‐doped nanocrystals that utilize sensitizer ions such as Yb^3+^ or Nd^3+^ to absorb NIR light and transfer the excitation energy to activator ions like Er^3+^, Ho^3+^, or Tm^3+^. This sequential energy transfer enables multi‐photon excitation, ultimately resulting in emissions in the visible or even UV spectral regions.^[^ ^45^, ^46^ ^]^ In conventional UCNPs, the lanthanide dopant concentrations are kept moderate, with sensitizer ion content below 30% and activator ions in the range of 0.1 to 2%.^[^ ^45^ ^]^

While UCNPs offer an appealing route to convert NIR photons into higher‐energy light, several challenges have limited their broader application in NIR‐driven photochemistry. First, the intrinsically low absorption cross‐sections of lanthanide ions^[^ ^47^ ^]^ limit upconversion efficiencies in the UV region and require careful nanostructural design to mitigate. To address these issues, several strategies have already been proposed, including host lattice engineering,^[^ ^48^, ^49^, ^50^ ^]^ dye sensitization,^[^ ^51^, ^52^, ^53^, ^54^ ^]^ and plasmonic coupling.^[^ ^55^, ^56^ ^]^ However, these approaches often involve compromises, such as photobleaching and non‐radiative losses, ultimately limiting the achievable enhancement. Second, the nanoscale dimensions of UCNPs make them especially vulnerable to surface‐mediated luminescence quenching,^[^ ^57^ ^]^ where excitation energy is lost through surface defects or non‐radiative interactions with solvent molecules. Through optimized dopant concentrations and surface passivation, these constraints can be partially overcome, enabling brighter emission and therefore improved catalytic performance. However, a comprehensive understanding of how particle architecture can be specifically adjusted to enhance UV emission for NIR‐to‐UV photocatalysis remains underdeveloped.

In this study, we demonstrate that efficient NIR‐driven photoreactions can be achieved under 980 nm excitation by carefully tuning the composition and architecture of UCNPs. We synthesized core‐shell NaYbF_4_(0.4% Tm)@NaYF_4_ nanoparticles, designing a fully sensitized lattice while maintaining low Tm^3+^ concentrations to minimize cross‐relaxation. This strategy led to a substantial increase in UV and blue upconversion emission. For comparison, we also synthesized conventional low doped UCNPs (NaYF_4_(25% Yb, 0.3% Tm)@NaYF_4_) to investigate the mechanisms underlying the observed enhancements. Our data indicate that reduced interionic distances in fully sensitized systems facilitate more efficient energy migration and transfer, resulting in markedly improved upconversion performance.

Recognizing the potential of this enhanced UV emission, and inspired by a few pioneering reports on NIR‐driven UV‐sensitive caged‐release systems,^[^ ^58^, ^59^, ^60^, ^61^, ^62^, ^63^ ^]^ (Scheme 1d), we hypothesized that our rationally engineered UCNPs could serve as effective photocatalysts for [2 + 2] photocycloaddition reactions by enabling singlet excitation of simple olefins under NIR irradiation (Scheme 1e). Instead of maximizing absolute brightness, our strategy focuses on enabling localized UV light generation through upconversion directly at the reaction site, using tissue‐penetrating near‐infrared excitation. This concept is particularly advantageous for photochemical reactions in sealed, turbid, or biological systems where conventional UV illumination is inefficient or impractical.

## Results and Discussion

To enhance upconversion efficiency in the UV spectral range under 980 nm excitation, we systematically optimized the composition and structure of UCNPs. Our goal was to overcome the key limitations associated with low absorption cross‐sections, energy migration losses, and surface‐mediated quenching, all of which reduce the performance of UCNPs in photochemical applications.^[^ ^64^, ^65^, ^66^ ^]^

**Figure 1:**
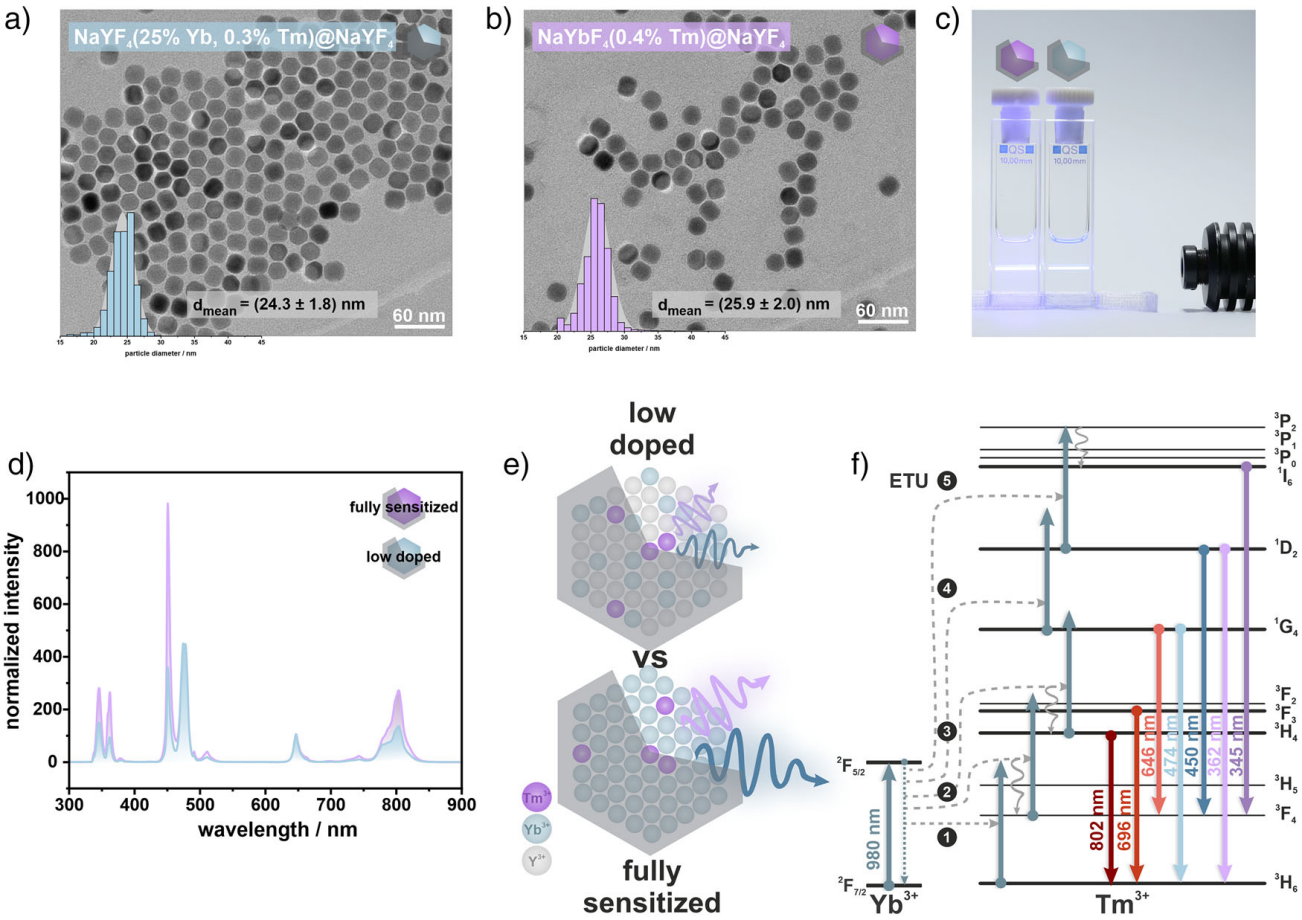
Characterization of low doped (NaYF_4_(25% Yb, 0.3% Tm)@NaYF_4_) and fully sensitized (NaYbF_4_(0.4% Tm)@NaYF_4_) UCNPs. TEM micrographs of a) NaYF_4_(25% Yb, 0.3% Tm)@NaYF_4_ (diameter (24.3 ± 1.8) nm) and b) NaYbF_4_(0.4% Tm)@NaYF_4_ (diameter (25.9 ± 2.0) nm) nanoparticles with corresponding size distribution histogram. c) Photograph showing dispersions of fully sensitized nanoparticles (left) and low doped nanoparticles (right) under 980 nm (cw) excitation. d) Luminescence spectra of both nanoparticle types in cyclohexane under 980 nm (cw) excitation at a power density of 150 W cm^−2^, normalized to the particle concentration. e) Schematic comparison of low doped and fully sensitized core‐shell nanoparticles. Purple wavy arrows denote UV emissions, and blue wavy arrows indicate blue emissions. Purple spheres represent Tm^3+^ ions, light blue spheres represent Yb^3+^ ions, and gray spheres represent Y^3+^ ions. f) Energy level diagram for Yb^3+^, Tm^3+^ co‐doped nanoparticles showing characteristic emissions up to five‐photon processes. Solid arrows indicate radiative energy transfers, dashed arrows denote energy transfer upconversion (ETU), and wavy arrows represent non‐radiative relaxations. The length of the gray‐blue solid arrows corresponds to the Yb^3+ 2^F_7/2_ → ^2^F_5/2_ energy gap, with energies adopted from Refs. [76, 77].

We identified that increasing the concentration of sensitizer ions is an effective strategy to enhance the absorption cross‐section of UCNPs, thereby improving their ability to capture NIR excitation light.^[^ ^67^, ^68^, ^69^ ^]^ To evaluate this effect, we compared low doped β‐NaYF_4_(25% Yb, 0.3% Tm) nanoparticles (22 ± 2 nm diameter, Figures S1a andS2) with fully sensitized β‐NaYbF_4_(0.4% Tm) nanoparticles (23 ± 2 nm diameter, Figures S1b and S2). As anticipated, the fully sensitized particles exhibited a fourfold increase in absorbance per particle (Figure S1c), confirming their superior excitation capacity under 980 nm irradiation. Surprisingly, however, despite this enhanced absorption, both nanoparticle types displayed comparable emission intensities. In fact, the low doped particles exhibited a slightly higher total emission, approximately 1.2 times greater, across the integrated spectral range from 300 to 900 nm (Figure S1d). This discrepancy is likely attributed to the reduced interionic distance between Yb^3+^ ions in NaYbF_4_ (0.26 nm versus 0.42 nm in low doped particles), which facilitates more efficient energy migration within the sensitizer sublattice. While beneficial for energy transfer, this enhanced migration also increases the probability of non‐radiative relaxation at the particle surface via interactions with solvent molecules, surface ligands, or defect sites.^[^ ^67^, ^70^, ^71^ ^]^ To mitigate these surface‐related losses, we equipped the nanoparticles with an optically inert shell of ∼1.5 nm thickness. This shell effectively suppressed surface quenching, resulting in a remarkable enhancement of luminescence intensity across the UV (345 nm and 362 nm), blue (450 nm), and NIR (802 nm) emission regions. Specifically, the emission at 345 nm increased by up to ≈ 210‐fold in fully sensitized particles and ≈ 90‐fold in conventionally doped ones. For the 362 nm emission, the enhancement reached ≈ 90‐fold and ≈ 20‐fold, respectively; for 450 nm, ≈100‐fold and ≈ 30‐fold; and for 802 nm, ≈14‐fold and ≈ 8‐fold (Figure 1c–e). While partial cation intermixing at the core‐shell interface cannot be ruled out, the observed luminescence enhancement upon shell growth strongly suggests a significant reduction of surface‐related quenching processes.^[^ ^72^, ^73^, ^74^ ^]^ Notably, the strongest enhancement occurs at 345 nm, corresponding to a five‐photon upconversion process (Figure 1f). The shorter Yb^3+^–Tm^3+^ interionic distance in the fully sensitized lattice (∼40% reduction) allows a greater number of Yb^3+^ ions to transfer excitation energy to a single Tm^3+^ activator. This increased donor density enhances energy transfer efficiency. Given the long‐lived nature of lanthanide energy levels, the presence of more energy donors accelerates the sequential energy transfer steps required to populate higher‐lying energy levels.^[^ ^75^ ^]^

**Figure 2:**
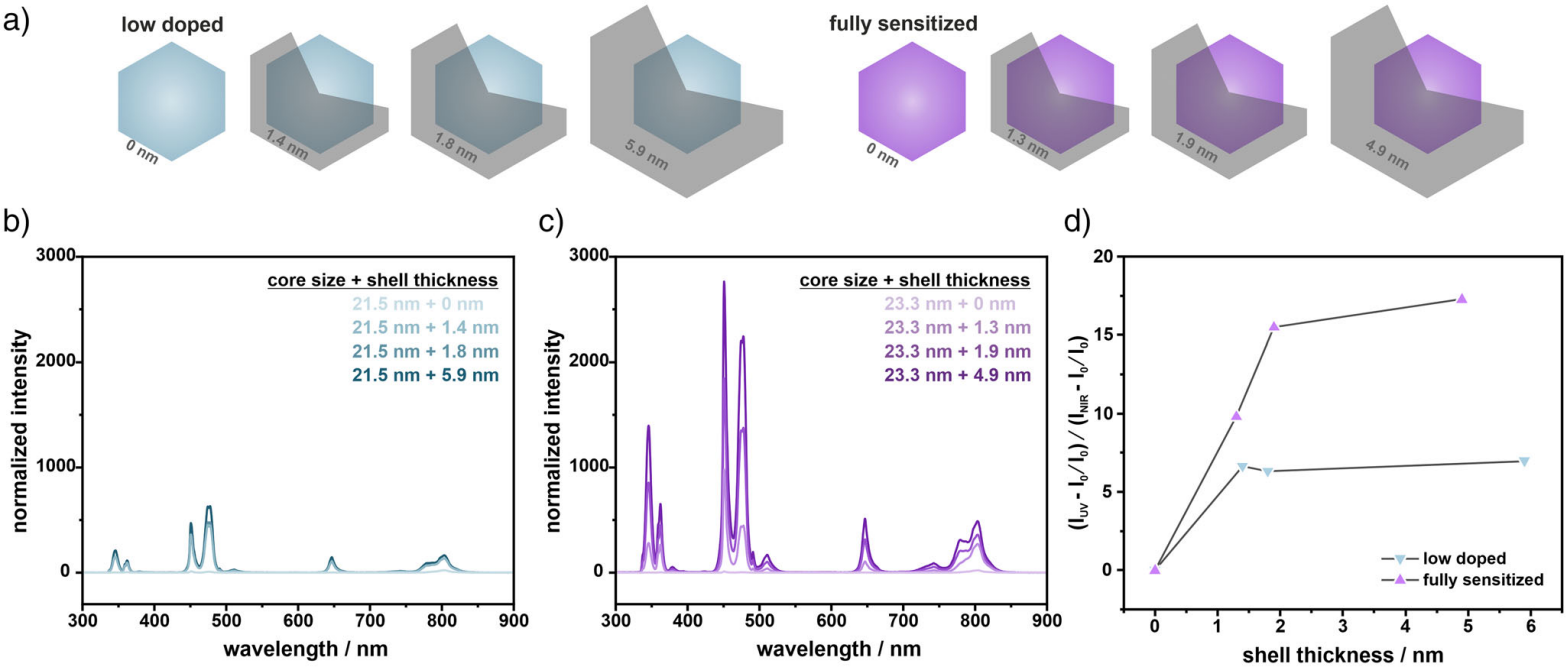
Influence of inert shell thickness on the luminescence properties of low doped and fully sensitized nanoparticles. a) Schematic representation of core‐shell nanoparticles with varying shell thicknesses for low doped and fully sensitized nanoparticles. b), c) Luminescence spectra of low doped NaYF_4_(25% Yb, 0.3% Tm)@NaYF_4_ and fully sensitized NaYbF_4_(0.4% Tm)@NaYF_4_ nanoparticles with different optically inert shell thicknesses. d) Relative change in UV‐to‐NIR intensity ratio (integration range 330 – 354 nm for UV and 765 – 851 nm for NIR) normalized to the emission intensity of the respective emission band of the core particles as a function of the optically inert shell thickness of both low doped and fully sensitized nanoparticles. All spectra were recorded in cyclohexane under 980 nm (cw) excitation at a power density of 150 W cm^−2^ and normalized to the particle concentration.

As an inert shell had a substantial impact on the emission intensities of UCNPs, we systematically investigated how shell thickness influences luminescence properties. To this end, we synthesized and characterized both fully sensitized and low doped core‐shell nanoparticles with shell thicknesses ranging from 1.5 to 5 nm (Figures 2 and S3). In low doped nanoparticles, a thin shell layer initially led to a significant enhancement in luminescence; however, further increases in shell thickness had only a minor effect on the overall emission intensity (Figure 2b). In contrast, fully sensitized nanoparticles exhibited a continuous increase in luminescence with increasing shell thickness (Figure 2c), indicating effective suppression of long‐range quenching processes.^[^ ^78^ ^]^

Since thulium is not highly susceptible to solvent quenching,^[^ ^79^ ^]^ the pronounced shell‐dependent effect is likely attributable to the high concentration of ytterbium ions. At elevated doping levels, Yb^3+^ ions are prone to surface quenching due to their efficient energy migration to the nanoparticle surface, where excitation energy is lost at defect sites.^[^ ^65^, ^71^, ^80^ ^]^ Although Yb^3+^ does not undergo strong solvent quenching like Er^3+^, its high energy migration within the lattice makes it particularly vulnerable to surface‐related losses.^[^ ^65^, ^80^ ^]^ Encapsulating the nanoparticles with a thick, optically inert shell effectively minimizes these losses, leading to a substantial increase in overall upconversion luminescence. The shell thickness also influenced the relative change in blue (450, 474 nm) and UV (345, 362 nm) emission compared to the NIR (802 nm) emission in fully sensitized versus low doped UCNPs (Figures 2d and S4). For both blue and UV emissions, a similar trend in relative emission ratios was observable. In low doped UCNPs, the blue and UV emissions experienced a stronger enhancement in emission intensity with the application of a thin inert shell layer compared to the NIR emission. However, with increasing shell thickness, the relative UV/blue‐to‐NIR emission reached a plateau with shell thicknesses above 1.5 nm. In contrast, for fully sensitized UCNPs, the relative UV/blue enhancement compared to the NIR enhancement steadily increased with increasing shell thicknesses. It plateaued with shell thicknesses exceeding 2 nm, which is consistent with the suppression of the quenching of migrated energy within the Yb^3+^ sublattice. At larger shell thicknesses, energy migration persists without being quenched, enabling more efficient excitation energy transfer to Tm^3+^. The short Yb^3+^–Tm^3+^ interionic distance (0.26 nm) promotes rapid energy transfer, thereby enhancing the population of higher‐energy states, such as the ^1^D_2_ level, which is responsible for the 450 nm emission (Figure 1f).

Accordingly, time‐dependent emission profiles for Yb^3+^ and Tm^3+^ in both fully sensitized and low doped UCNPs revealed that luminescence lifetimes increase with shell thickness (Figures S5–S8). However, across all shell thicknesses and emission wavelengths of both Yb^3+^ and Tm^3+^, fully sensitized nanoparticles exhibited shorter luminescence lifetimes compared to their low doped counterparts, despite achieving up to 4.5 times higher overall emission intensities (integration range 300 – 900 nm). This trend reflects the complex interplay of multiple mechanisms. On the one hand, it can be attributed to the inverse relationship between the absorption cross‐section and excited‐state lifetime, where increasing the number of absorbing ions (Yb^3+^) enhances excitation efficiency but shortens the excited‐state lifetime.^[^ ^70^ ^]^ As surface‐related quenching is expected to be effectively suppressed at high shell thicknesses,^[^ ^81^ ^]^ particularly the shortening of the Yb^3+^ emission decay profile suggests more efficient energy transfer from Yb^3+^ to Tm^3+^. On the other hand, the observed lifetime shortening for Tm^3+^ emissions may also be influenced by non‐radiative processes such as back energy transfers from Tm^3+^ to Yb^3+^ or cross‐relaxations. Therefore, the reduced lifetimes observed in fully sensitized UCNPs result from a combination of efficient energy transfer from Yb^3+^ to Tm^3+^ and back energy transfer, as observed luminescence lifetimes represent the overall upconversion kinetics rather than intrinsic excited‐state lifetimes.^[^ ^82^, ^83^, ^84^ ^]^

The spectral analysis of fully sensitized and low doped nanoparticles further reveals changes in the emission ratio between the 450 and 474 nm peaks. To investigate the underlying mechanism, we recorded luminescence spectra under non‐steady‐state excitation with varying pulse lengths but at a constant duty cycle (Figure S9). This approach allows differentiation between energy pathways and helps to identify the contribution of higher‐lying energy states to specific emission bands.^[^ ^69^, ^85^ ^]^ As expected, reducing the pulse width led to a general decrease in the overall emission intensity for both fully sensitized and low doped nanoparticles (Figure S9a,c). However, in low doped UCNPs, emissions from higher‐energy levels, such as 450 and 362 nm (both ^1^D_2_ energy level) and 345 nm (^1^I_6_ energy level), declined sharply when the pulse width was shortened from 1.5 ms to 150 µs, becoming undetectable below 750 µs (Figure S9c,d). This sharp reduction suggests that the population of these states requires prolonged excitation, likely due to significant energy mismatches in non‐resonant Yb^3+^–Tm^3+^ energy transfer steps, particularly from ^1^G_4_ to ^1^D_2_ transitions. In contrast, all emission bands remained detectable across the full pulse width range in fully sensitized nanoparticles (Figures S9a,b). While the 345 nm emission exhibited a strong dependence on pulse width, the emissions at 362 nm and 450 nm were relatively stable, suggesting the presence of an alternative population pathway. This behavior can be attributed to Tm^3+^–Tm^3+^ energy transfer processes that populate the ^1^D_2_ state, responsible for the 362 nm and 450 nm emissions. These interactions become more probable in fully sensitized UCNPs due to the shorter average Tm^3+^–Tm^3+^ distance (1.6 nm) compared to that in low doped systems (1.8 nm). Detailed mechanisms of these energy transfer processes are summarized in Figure S10.^[^ ^86^, ^87^ ^]^ Temporal luminescence response curves (Figures S11 and S12) further support these findings. Low doped nanoparticles exhibited longer rise times for all emission bands, likely reflecting a cooperative effect involving fast Yb^3+^–Tm^3+^ energy transfer and slower Tm^3+^–Tm^3+^ interactions that gradually populate higher‐energy states. Interestingly, on the other hand, in fully sensitized UCNPs, the 450 nm (^1^D_2_) emission exhibited a slightly prolonged rise time compared to the 345 nm (^1^I_6_) emission. This delay may indicate a secondary, slower population pathway consistent with Ln^3+^–Ln^3+^ energy transfer processes (Figure S10).

These results, therefore, underscore the significance of the ^1^G_4_ level (responsible for 474 and 646 nm emissions) as an intermediate state in the population pathway of Tm^3+^. This role is further supported by power density‐dependent luminescence measurements (Figure S13). Together, these findings highlight how sensitizer concentration and interionic spacing influence UCNP emission dynamics, offering valuable insights for applications that require controlled population of high‐energy excited states, such as in photochemical reactions.

**Figure 3:**
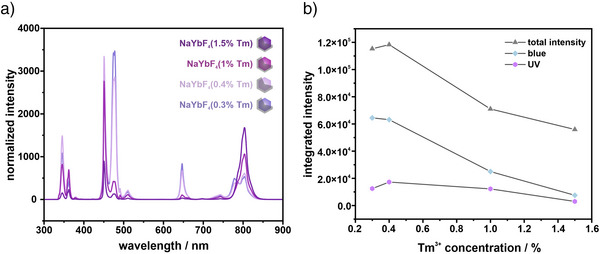
Effect of Tm^3+^ concentration on the luminescence properties of fully sensitized core‐shell nanoparticles. a) Luminescence spectra of  NaYbF_4_(Tm)@NaF_4_ core‐shell nanoparticles with varying Tm^3+^ concentrations (0.3%, 0.4%, 1%, and 1.5%). b) Integrated intensity of blue (integration range 435 – 491 nm) and UV (integration range 330 – 354 nm) emissions, as well as the total luminescence intensity (integration range 300 – 900 nm) as a function of Tm^3+^ concentration. All luminescence spectra were recorded in cyclohexane under 980 nm (cw) excitation at a power density of 150 W cm^−2^ and normalized to the particle concentration.

Since small reductions in Tm^3+^–Tm^3+^ interionic distances have been shown to enhance blue and UV emissions in fully sensitized UCNPs, we further investigated the effect of thulium concentration on luminescence properties to identify the optimum activator content for maximizing emission in the high‐energy spectral region (Figures 3, S14, and S15). Increasing the Tm^3+^ concentration initially led to an increase in both overall emission intensity and the relative contribution of blue and UV bands (Figures 3 and S15). However, when the Tm^3+^ doping exceeded 1%, the overall intensity, as well as the emissions in the blue and UV regions declined (Figure 3b). At higher doping levels (e.g., 1.5% Tm^3+^), the reduced Tm^3+^–Tm^3+^ distance (1.0 nm) promotes deleterious cross‐relaxation processes. These processes depopulate the intermediate ^1^G_4_ state (Figure S10), thereby outweighing the benefits of increased energy transfer and ultimately reducing overall brightness. Overall, a doping concentration of 0.4% Tm^3+^ has proven to be optimal to take advantage of Tm^3+^–Tm^3+^ energy transfers.

Consequently, as interionic distances decrease, both beneficial and detrimental energy pathways become more probable. In addition to cross‐relaxation, another competing process is back‐energy transfer, in which excitation energy is transferred from an activator ion (Tm^3+^) back to a sensitizer ion (Yb^3+^). While this phenomenon is well‐documented in Er^3+^‐doped UCNPs with high Yb^3+^ content,^[^ ^67^, ^69^, ^88^ ^]^ it has been less frequently reported in Tm^3+^‐doped systems.^[^ ^75^, ^89^ ^]^ Nevertheless, the probability of back‐energy transfer increases with decreasing Yb^3+^–Tm^3+^ distances, particularly at elevated doping levels. To investigate this effect, we directly excited Tm^3+^ ions at 808 nm in both fully sensitized and low doped nanoparticles (Figure S16). In fully sensitized UCNPs, emission bands were observed at 474, 646, and 696 nm, whereas in low doped nanoparticles, the emission was dominated by the 474 nm band. The 474 nm and 646 nm emissions can be accessed via direct two‐photon excited state absorption, whereas the 696 nm emission requires a two‐photon energy transfer from Yb^3+^ to Tm^3+^ (Figure S16b).^[^ ^90^ ^]^ Therefore, the presence of the 696 nm band in fully sensitized UCNPs suggests significant back‐energy transfer from Tm^3+^ to Yb^3+^. Furthermore, due to the considerable energy mismatch between the 808 nm excitation and the ^3^H_4_ to ^1^G_4_ transition, the population of ^1^G_4_ (emitting at 474 nm and 646 nm) is less efficient via excited‐state absorption. This contributes to lower emission intensities in low doped nanoparticles under 808 nm excitation, further highlighting the sensitivity of emission behavior to the dopant configuration. Although back‐energy transfer is often considered a quenching mechanism, Li Xu et al.^[^ ^90^ ^]^ proposed an energy cycling scheme in which back‐energy transfer, followed by subsequent energy transfer upconversion, can enhance the overall efficiency of sensitizer‐activator co‐doped systems. In this context, such a mechanism may also be advantageous in fully sensitized UCNPs by harnessing back‐energy transfer to boost NIR emission at 802 nm, thereby improving overall photon utilization.

A detailed understanding of dopant interaction, energy migration, and back‐energy transfer in fully sensitized UCNPs provides a foundation for optimizing their performance in NIR‐driven photoreactions. By controlling dopant concentrations, implementing effective surface passivation, and leveraging energy cycling mechanisms, UCNPs can be rationally engineered for applications that demand efficient photon conversion and enhanced high‐energy emission.

**Scheme 2:**
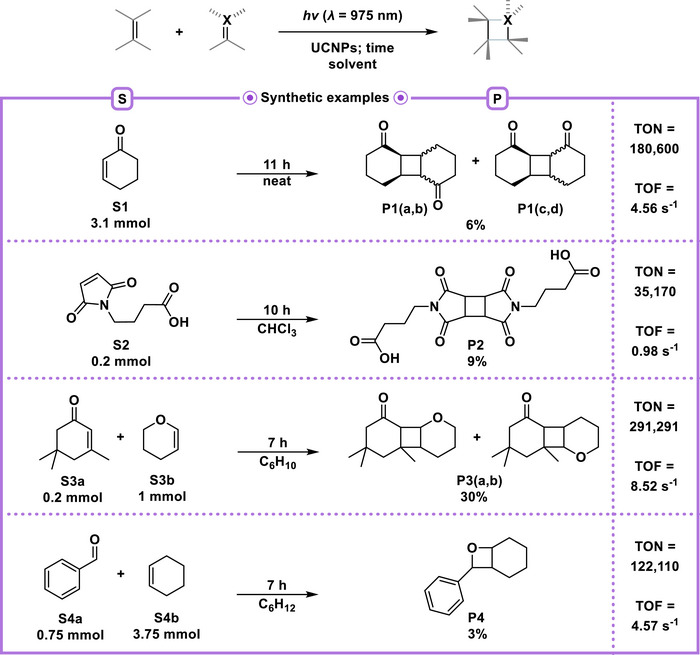
Examples of NIR‐light‐driven [2 + 2] photocycloadditions using UCNPs as catalysts. Isolated yields of the desired products are reported unless noted otherwise. (Section 1.7 in the Figures S19–S31).

With the optimized UCNPs in hand, we investigated the dimerization of cyclohexenone **S1** via UVA‐promoted [2 + 2] photocycloaddition (Scheme 2). The reaction was conducted under neat conditions, utilizing continuous‐wave (cw) laser diode irradiation with an average output power of 1 W. The beam was focused using a lens system to maximize intensity (Figure S17), resulting in an average beam diameter of approximately 1.5 mm^2^. Prolonged excitation of the UCNP dispersion at 980 nm resulted only in a negligible temperature increase, suggesting no risk of thermally induced side effects (Figure S18). A low catalytic loading of ∼2.27 × 10^14^ nanoparticles per mL was used. After 6 h of irradiation, the reaction yielded approximately 0.66% (0.034 mmol) of the desired diastereomeric product mixture **P1(a‐d)** from 1 mL (10.34 mmol) of starting material. While the obtained yield in neat solvent appears to be relatively low, the catalyst demonstrated high turnover numbers (TON) of ∼90,167 and turnover frequencies (TOF)^[^ ^91^ ^]^ of 4.17 s^−1^, surpassing those of commonly used photosensitizers and photoredox catalysts in synthesis.^[^ ^92^, ^93^ ^]^

We were pleased to observe that increasing the catalytic loading (up to ∼1.07 × 10^15^ particles per mL) and extending the reaction time to 11 h significantly improved the yield to 6% and the quantity of products **P1(a‐d)** (approximately 0.1 mmol) from 0.3 mL of starting material **S1**. This condition also resulted in an increase in the TON to 180,600 and a slight increase in the TOF to 4.56 s^−1^. In the case of 4‐(2,5‐dioxo‐2,5‐dihydro‐1H‐pyrrol‐1‐yl)butanoic acid **S2**, we demonstrated that even at a lower substrate concentration of approximately 0.67 mol L^−1^ in chloroform solution, substrates successfully undergo the [2 + 2] photodimerization, resulting in an approximate yield of 9% (∼0.01 mmol) of a single diastereomer of product **P2**, with a TON of 35170 and a TOF of 0.98 s^−1^. The UCNPs could also be recovered by centrifugation from the reaction mixture with recoveries of approximately 40‐50%. Manual handling and inefficiencies in small‐scale centrifugation primarily caused the reduced particle recovery observed. Automated separation strategies, such as tangential flow filtration, may greatly improve the recovery in future scalable processes. Next, we applied the optimized UCNP system to the [2 + 2] photocycloaddition of isophorone **S3a** and the electron‐rich olefin 3,4‐dihydro‐2H‐pyran **S3b**, yielding up to 30% (0.06 mmol) of the diastereomeric products **P3(ab)**. This transformation achieved a considerably high TON of 291,291 and a TOF of 8.52 s^−1^. UCNP‐catalysis was used to promote a Paternò‐Büchi reaction, wherein benzaldehyde **S4a** was activated via the emission of the UCNPs, generating a singlet excited species that subsequently reacted with cyclohexene **S4b**. This led to the formation of the corresponding single major diastereomer oxetane **P4** with a yield of approximately 3% (0.02 mmol), achieving a high TON of 122,110 and a TOF of 4.57 s^−1^.

All reactions yielded products on a multi‐milligram scale, which represents a significant achievement given the challenges typically associated with upconversion‐driven processes.^[^ ^24^ ^]^ However, calculated yields remain low despite high TON and TOF of the UCNPs due to the neat reaction conditions used in many examples. Without further surface modifications, the catalyst can only be used in nonpolar or weakly polar media to avoid aggregation and precipitation. To benchmark our photocatalytic system, we compared its performance with representative UC approaches from recent literature, focusing on excitation/emission wavelengths, energy loss, excitation intensity, and light source requirements (Table S2).^[^ ^24^, ^94^, ^95^ ^]^ Our system converts 980 nm excitation into 345 nm UV emission via a five‐photon process, with an energy loss of 43%, comparable to TTA‐UC systems (25%–40%). Unlike TTA‐UC, it combines the deep tissue penetration of NIR excitation with direct UV output. Compared to SHG, it operates at much lower excitation intensities (continuous‐wave laser, 1.5 × 10^2^ W cm^−2^ versus > 10^11^ W cm^−2^). Direct two‐photon absorption approaches remain impractical for UV generation, as suitable TPA dyes are unavailable.

## Conclusion

In conclusion, this study demonstrates the use of UCNPs as catalysts for NIR‐driven UV photochemistry. Tailored for that, we developed a class of fully sensitized UCNPs by fully populating the host lattice with the sensitizer Yb^3+^ and maintaining low concentrations of the activator Tm^3+^. We achieved exceptional upconversion efficiencies in the UV and blue spectral regions. We further enhanced these emissions by systematically optimizing the thickness of the inert shell to minimize surface‐related quenching.

Our optimized NaYbF_4_:Tm@NaYF_4_ UCNPs demonstrated substantial improvements in photophysical properties compared to conventional low doped systems. These improvements include enhanced excitation efficiency, increased energy transfer rates, and higher‐order photon upconversion, most notably in the five‐photon transition at 345 nm. We also established mechanistic insights into energy migration, back‐energy transfer, and the role of interionic distances in determining UCNPs luminescence dynamics. This understanding not only advances the fundamental knowledge but also establishes a framework for the rational design of UCNPs tailored for efficient NIR‐to‐UV conversion.

These findings prompted us to employ these UCNPs as highly dispersed heterogeneous photocatalysts^[^ ^96^ ^]^ for NIR‐triggered [2 + 2] cycloadditions and Paternò–Büchi reactions, achieving turnover numbers exceeding 290,000 and turnover frequencies as high as 8.52 s^−1^. The values by far exceed those reported for classic photosensitizers and represent a significant step toward practical NIR‐to‐UV photocatalysis. Additionally, a simple recovery procedure, such as centrifugation, was demonstrated to be feasible, underscoring the catalyst's potential for reuse. The use of more sophisticated separation methods is expected to improve the recycling of UCNPs in photocatalytic reactions.

Future studies could also investigate more environmentally friendly solvent systems, microwave‐assisted synthesis, or flow reactors to improve sustainability and cost‐effectiveness. While the current system demonstrates excellent performance for small‐scale photocatalysis, future efforts should investigate the scalability and cost‐effectiveness of UCNP production compared to conventional UVB‐based methods. Flow reactors may provide an adaptable platform to transfer NIR‐driven UCNP photocatalysis to an application beyond the research laboratory. Furthermore, further surface engineering of UCNPs could improve ligand density, upconversion efficiency, and dispersion in complex media. However, our results pave the way for the rational design of next‐generation UCNP‐based catalysts that bridge the gap between NIR excitation for deep tissue/reaction mixture penetration and high‐energy UV photochemistry. Although the present excitation intensity exceeds biocompatibility limits, in situ UV generation via NIR excitation offers a promising route for future biomedical and confined‐environment applications.

We expect that advances in surface functionalization and biocompatibility will provide access to applications in aqueous and polar solvents, broadening the potential of UCNPs in synthetic photochemistry, bioorthogonal reactions, and live‐cell labeling.

## Supporting Information

The authors have cited additional references within the Supporting Information.^[^ ^24^, ^67^, ^94^, ^95^, ^97^, ^98^, ^99^, ^100^, ^101^, ^102^ ^]^

## Conflict of Interests

The authors declare no conflict of interest.

## Supporting information



Supporting Information

## Data Availability

The data that support the findings of this study are openly available in RADAR4Chem at https://doi.org/10.22000/enaah80uua1yrjs9.
