# Supplementary material for: Fully Sensitized Upconversion Nanoparticles as Efficient Catalysts for NIR‐Driven UV Photochemistry
Source: Angew Chem Int Ed Engl. 2025 Sep 16;64(46):e202511247. doi: 10.1002/anie.202511247 (PMC12603989; doi:10.1002/anie.202511247)
Supplement: Supplementary file 1 — Supporting Information [file ANIE-64-e202511247-s001.pdf]

Supporting Information

Fully Sensitized Upconversion Nanoparticles as Efficient Catalysts for NIR-Driven UV Photochemistry

Naomi Weitzel[1,†], Armaz Tsutskiridze[2,†], Julia Bramowski[1], Burkhard König[2,*], Thomas Hirsch[1,*]

†) contributed equally

*) corresponding authors

1) Institute of Analytical Chemistry, Chemo- and Biosensors, University of Regensburg, 93040 Regensburg, Germany

2) Institute of Organic Chemistry, University of Regensburg, 93040 Regensburg, Germany

Table of Contents

1 Experimental Details 3
1.1 Chemicals 3
1.2 Characterization 3
1.3 Preparation of Upconversion Nanoparticles 6
1.3.1 Synthesis of Core Nanoparticles 6
1.3.2 Synthesis of Core-Shell Nanoparticles 7
1.3.3 Ligand Exchange for Surface Modification of Nanoparticles 9
1.4 Calculation of Nanoparticle Concentrations 9
1.5 Calculation of Interionic Distances 10
1.6 Laser Diode Setup for Indirect Excitation of Photosensitive Reactions 10
1.7 Photocatalytic Reactions under NIR excitation 10
1.7.1 Dimerization of 2-Cyclohexenone 11
1.7.2 Dimerization of 4-(2,5-Dioxo-2,5-Dihydro-1H-Pyrrol-1-yl)Butanoic Acid 12
1.7.3 2+2 Photocycloaddition between 3,5,5-Trimethylcyclohex-2-En-1-One and 3,4-Dihydro-2H-Pyran 13
1.7.4 Paternò–Büchi Reaction Between Cyclohexene and Benzaldehyde 13
1.8 Preparation of Substrates under Direct UVA Irradiation 14
1.8.1 P1(a-d) 14
1.8.2 4,4'-(1,3,4,6-Tetraoxooctahydrocyclobuta[1,2-c:3,4-c']Dipyrrole-2,5-diyl)Dibutyric Acid (P2) 15
1.8.3 P3(a-b) 15
1.8.4 Endo-8-Phenyl-7-Oxabicyclo[4.2.0]Octane (P4) 16
2 Supporting Data and Figures 17
3 NMR Spectra 37
References 48

<u>Primary research data is available at:</u>

https://radar4chem.radar-service.eu/radar/en/dataset/enaah80uua1yrjs9?token=XboUSUwHgedNMRHXDNxP

1 Experimental Details

1.1 Chemicals

Ytterbium chloride hexahydrate ($YbCl_3 \cdot 6\ H_2O$, ≥ 99.9%) and yttrium chloride hexahydrate ($YCl_3 \cdot 6\ H_2O$, ≥ 99.9%) were acquired from *Treibacher Industrie AG*. Thulium chloride hexahydrate ($TmCl_3 \cdot 6\ H_2O$, ≥ 99.9%), nitrosyl tetrafluoroborate ($NOBF_4$, 95%), chloroform-*d* ($CDCl_3$, 99.8%), and sodium oleate (82%) were purchased from *Sigma Aldrich*. Oleic acid and octadecene, both of analytical grade (≥ 90%), were from *Alfa Aesar*. Sodium hydroxide (NaOH, 98%), cyclohexene (≥ 99%), benzaldehyde (≥ 99.5%), and ammonium fluoride (NH_4F, 98%) were obtained from *Merck*. Cyclohexane (analytical reagent grade), chloroform (≥ 99.8%), dimethyl formamide (DMF, ≥ 99.5%), methanol (MeOH, ≥ 99.9%), isophorone (97%), and sulfuric acid (H_2SO_4, ≥ 95%, w/w) were purchased from *Fisher Scientific*. Nitric acid (HNO_3, 65%, w/v) was acquired from *VWR Chemicals*. A multielement calibration standard containing Y^{3+}, Yb^{3+}, Er^{3+}, Tm^{3+}, Nd^{3+}, Gd^{3+}, each 1000 mg mL^{-1} (dissolved in 1.5 M HNO_3) was obtained from *Bernd Kraft*. 2-Cyclohexen-1-one (>96%), 1,4-bis(trifluoromethyl)benzene (>99%), and 4-maleimidobutryric acid (>98%) were obtained from *TCI*. Chloroform (extra dry over molecular sieve, stabilized) was purchased from *AcroSeal*. Dimethylsulfoxide-d_6 (DMSO-d_6, 99.8%) was acquired from *Deutero*. 3,4-Dihydro-2H-pyran (99%) was bought from *ABCR*. If not stated otherwise, all other commercially available organic chemicals and solvents were of the highest commercial quality or analytical grade and used without further purification. All photochemical reactions were conducted in dried and degassed solvents. Solvents for column chromatography were used without further purification. Commercially available starting materials and reagents were purchased and checked for purity by GC-MS and/or ^1H NMR spectroscopy and used without further purification.

1.2 Characterization

Transmission electron microscopy (TEM) was performed using a Philips CM12 microscope operating at 120 kV. Samples were prepared by dropping 20 µL of a nanoparticle solution (1.5 mg mL^{-1}) onto a 400-mesh copper grid. Particle size analysis was conducted automatically using *Fiji* (Version 1.54p), with at least 1,000 nanoparticles counted. *Origin 2024b* was used to create particle-size-distribution histograms and their corresponding Gaussian distribution.

Concentration analysis was performed *via* inductively coupled plasma optical emission spectroscopy (ICP-OES) using a SPECTROBLUE FMX36 (*Spectro*), calibrated with a multielement standard. For sample analysis, 10 µL of the nanoparticle dispersion was dried, dissolved in 0.5 mL H_2SO_4 (95% w/w), and diluted with 9.5 mL of 1.5M HNO_3.

Powder X-Ray diffraction (P-XRD) was carried out using a STOE STADI P diffractometer equipped with a Dectris Mythen 1K detector. Monochromatic $Cu_{K\alpha}$ radiation (λ = 1.54065 Å) was used, with a diffractogram resolution of 0.005° (2θ).

Absorbance spectra were recorded using a Cary 3500 photometer (*Agilent*) with excitation ranges from 800 – 1100 nm. Luminescence spectra were obtained using both a custom-built setup and commercial spectrofluorometers. The custom-built setup utilizes a *Qmini* UV/Vis spectrometer (*Broadcom*), coupled to *Waves* software, in a 90° configuration with a 980 nm, 200 mW continuous-wave (cw) laser module (*Picotronic*). Excitation light was filtered using a short-pass filter (cut-off: 860 nm) and a bandpass filter (cut-off: 900 nm) (*Thorlabs*). For pulsed excitation experiments, a FS5 spectrophotometer with a 980 nm, 2 W pulsed laser module (adjustable pulse length) was used (*Edinburgh Instruments*). For direct excitation of Tm^{3+}, additional measurements were recorded using FLS980 spectrophotometer equipped with an 808 nm, 8 W laser module (*Edinburgh Instruments*).
Note: In the custom-built luminescence set-up, no correction of the spectral sensitivity of the *Qmini* detector system was performed. All spectra were recorded under identical instrumental settings and are discussed only in the context of relative intensity differences between samples of matched concentrations and particle sizes. For comparison, the correction factors for the main emission bands of Yb^{3+}, Tm^{3+} doped UCNPs are denoted in Table **S1**.

Table S1: Correction factors for selected emission wavelengths of the Qmini detector.

Emission Wavelength	Correction Factor
802 nm	3.7
646 nm	1.1
474 nm	0.8
450 nm	0.8
362 nm	1.8
345 nm	2.2

Luminescence decay measurements were conducted on a FS5 spectrophotometer (*Edinburgh Instruments*) equipped with 980 nm, 2 W laser module. Indirect excitation of photosensitive reactions under 980 nm excitation were performed using a focused 980 nm, 3 W cw laser module. (*Roithner Lasertechnik*). Monitoring of the temporal development during 980 nm, cw laser excitation was performed using a P2 Pro thermographic camera (*Infiray*).

Gas chromatography (GC) measurements were performed on a GC 7890 (*Agilent Technologies*). Data acquisition and evaluation were done with *Agilent Chem Station Rev.C.01.04*. GC–MS measurements were performed on a 7890A GC system (*Agilent Technologies*) with an Agilent 5975 MSD Detector. Data acquisition and evaluation were done with *MSD Chem Station E.02.02.1431*. A capillary column HP-5MS/30 mx 0.25 mm/0.25 μM film and helium as carrier gas (flow rate of 1 mL min^{-1}) were used. The GC oven temperature program was adjusted as follows: the initial temperature (40 °C) was kept for 3 minutes, and the temperature was increased at a rate of 15 °C min^{-1} over 16 minutes until 280 °C was reached and kept for 5 minutes, the temperature was again increased at a rate of 25 °C min^{-1} over seconds until the final temperature (300 °C) was reached and kept for 5 minutes.

Column chromatography was performed in the *Biotage* Isolera One 3.0 or on normal-grade silica gel (SiO_2, 60 Å) in a cylindrical glass column with isocratic elution (composition of the eluent is given for each experiment). Chromatography solvents were distilled before use.

Analytical thin-layer chromatography (TLC) was performed on silica gel-coated alumina plates (MN pre-coated TLC-sheets *ALUGRAM*® Xtra SIL G/UV254). Visualization was done by UV light (254 nm or 366 nm) or by staining with $KMnO_4$.

All NMR spectra of the isolated compounds were measured at room temperature using a *Bruker* Avance 400 (400 MHz for ^{1}H, 101 MHz for $^{13}C\{^{1}H\}$ and 377 MHz for $^{19}F\{^{1}H\}$) NMR spectrometer. All chemical shifts are reported in δ-scale as parts per million [ppm] (multiplicity, coupling constant J, number of protons). ^{1}H NMR chemical shifts are reported relative to TMS and were referenced via residual proton resonances of the corresponding deuterated solvent ($CDCl_3$: 7.26 ppm, DMSO-d_6: 2.50 ppm) and $^{13}C\{^{1}H\}$ NMR spectra are reported relative to TMS and were referenced *via* the carbon signals of the deuterated solvent ($CDCl_3$: 77.16 ppm, DMSO-d6: 39.52 ppm). Coupling constants *J* are given in Hertz [Hz]. Abbreviations used for signal multiplicity: ^{1}H NMR: s = singlet, d = doublet, dd = doublet of doublets, ddd = doublet of doublets of doublets, dt = doublet of triplets,

t = triplet, td = triplet of doublets, q = quartet, m = multiplet, and br. = broad; ^{13}C-NMR: (1°) = primary, (2°) = secondary, (3°) = tertiary, (4°) = quaternary carbon. Spectra were analyzed using *Bruker Topspin 4.1.4*.

High-resolution mass spectra (HRMS) were obtained from the central analytical mass spectrometry facilities of the Faculty of Chemistry and Pharmacy, Regensburg University, and are reported according to the 2013 IUPAC recommendations. All mass spectra were recorded on a Finnigan MAT 95, Thermo Quest Finnigan TSQ 7000, Finnigan MATSSQ 710 A, or Agilent Q-TOF 6540 UHD instrument.

1.3 Preparation of Upconversion Nanoparticles

1.3.1 Synthesis of Core Nanoparticles

Fully sensitized upconversion nanoparticles were synthesized following a modified protocol based on Schroter *et al.*[1] For a 5 mmol batch of $NaYbF_4$(0.3% Tm) nanoparticles, $YbCl_3$· 6 H_2O (4.985 mmol) and $TmCl_3$ · 6 H_2O (0.015 mmol) were dissolved in a small volume of methanol and transferred to a three-neck round-bottom flask flushed with nitrogen. Nanoparticles with varying doping ratios were synthesized by adjusting the molar ratios of the desired $LnCl_3$ · 6 H_2O precursors. Subsequently, oleic acid (41.875 mL) and octadecene (50 mL) were added, and the mixture was heated to 110 °C under vacuum for 1.5 h. After cooling to room temperature, sodium oleate (10.625 mmol), ammonium fluoride (25 mmol), and octadecene (21.875 mL) were added. The solution was subjected to vacuum for 45 min, followed by three nitrogen purges to ensure an inert atmosphere. The reaction mixture was then heated to 315 °C under reflux at a rate of 16 °C min^{-1} and maintained at this temperature for 30 min. After rapid cooling to room temperature, nanoparticles were precipitated by ethanol addition and collected *via* centrifugation at 3,850 *g* for 15 min. The particles were purified by three washing cycle with cyclohexane and excess ethanol, followed by removal of aggregates via centrifugation at 1,000 *g* for 3 min. The purified nanoparticles were stored in cyclohexane at 6 °C.

Low doped nanoparticles were synthesized following a modified protocol based on Wilhelm *et al.*[2] For a 5 mmol batch of $NaYF_4$(15% Yb, 0.3% Tm), YCl_3 · 6 H_2O (3.735 mmol), $YbCl_3$ · 6 H_2O (1.25 mmol) and $TmCl_3$ · 6 H_2O (0.015 mmol) were dissolved in a small volume of methanol and transferred to a three-neck round-bottom flask under nitrogen atmosphere. Subsequently, oleic acid (40 mL) and octadecene (75 mL) were added, and the mixture was heated to 140 °C under vacuum for 30 min to remove residual solvents. After cooling to room

temperature, a methanolic solution of sodium hydroxide (12.5 mmol) and ammonium fluoride (20 mmol) was added. To eliminate excess methanol and crystal water, the solution was heated to 120 °C and maintained for 20 min. The reaction mixture was heated to 325 °C at a rate of 16 °C min^{-1} and kept at this temperature for 25 min, followed by a rapid cooling to room temperature. Purification was performed as described above. The final nanoparticles were stored in cyclohexane at 6 °C.

1.3.2 Synthesis of Core-Shell Nanoparticles

For the synthesis of core-shell nanoparticles, a sacrificial shell precursor was prepared as an intermediate step. For a 20 mmol batch of cubic $NaYF_4$ nanoparticles, $YCl_3 \cdot 6\,H_2O$ was dissolved in methanol and transferred to a three-neck round-bottom flask under a nitrogen atmosphere. Subsequently, oleic acid (160 mL) and octadecene (300 mL) were added, and the mixture was heated to 160 °C under vacuum for 30 min. After cooling to room temperature, a methanolic solution of sodium hydroxide (50 mmol) and ammonium fluoride (80 mmol) was added. The solution was then heated to 120 °C and maintained for 20 min to eliminate excess methanol and any remaining crystal water. The reaction mixture was heated to 240 °C at a rate of 16 °C min^{-1} and kept at this temperature for 30 min, followed by rapid cooling to room temperature. The purification was performed according to the already described protocol. The cubic shell precursor nanoparticles were stored in cyclohexane at 6 °C.

To achieve precise and reproducible shell thickness, the required amount of cubic shell precursor was calculated as follows:

The volume of one core nanoparticle (V_{core}) was calculated using equation (1), whereas the volume of one cubic shell precursor (V_{shell}) was calculated using equation (2). For the hexagonal $NaLnF_4$ nanoparticles <35 nm, a quasi-spherical morphology was assumed.

$$V_{core} = \frac{4}{3} \cdot \pi \cdot \left(\frac{d_{core}}{2}\right)^3 \tag{1}$$

$$V_{shell} = \frac{4}{3} \cdot \pi \cdot \left(\frac{d_{core} + d_{shell}}{2} - \frac{d_{core}}{2}\right)^3 \tag{2}$$

With d_{core} being the core diameter derived from TEM studies and d_{shell} being the desired shell thickness.

Using the volume of the core particles and the volume of the inert shell of a defined thickness the molar ratio of the core particle solution and the cubic shell precursor ($n_{core}\ n_{shell}^{-1}$) can be retrieved from equation (3). Applying a defined molar amount of core particles (n_{core}, *e.g.*, 0.5 mmol) the respective molar amount of shell precursor (n_{shell}) can be derived from equation (3).

$$\frac{n_{core}}{n_{shell}} = \frac{\rho_{core} \cdot V_{core} \cdot M_{shell}}{\rho_{shell} \cdot V_{shell} \cdot M_{core}} \tag{3}$$

With ρ_{core} being the theoretical density of the core nanoparticle, ρ_{shell} being the theoretical shell density, M_{core} being the calculated molar mass of one core particle and M_{shell} being the calculated molar mass of one cubic shell precursor particle.

The volume of the shell precursor ($V_{precursor}$) needed was calculated according to equation (4) using the molar amount of shell precursor n_{shell}, the calculated molar mass of one cubic shell precursor particle M_{shell}, and the mass concentration β_{shell} determined by ICP-OES measurements.

$$V_{precursor} = \frac{n_{shell} \cdot M_{shell}}{\beta_{shell}} \tag{4}$$

For a 1.5 nm shell thickness, 0.5 mmol of core particles and the calculated amount of cubic shell precursor (0.345 mmol) were each dissolved in cyclohexane and transferred to separate round-bottom flasks containing to oleic acid (2.5 mL) and octadecene (2.5 mL) under nitrogen atmosphere. Excess cyclohexane was removed by heating the solutions to 100 °C under vacuum for 30 min. The core particle dispersion was then heated to 315 °C at a rate of 16 °C min^{-1}, while the shell precursor dispersion was maintained at 100 °C. During heating, 0.5 mL aliquot of the shell precursor solution was injected into the core particle solution. Once the temperature reached 315 °C, additional shell precursor was incrementally added every 10 min. After the final injection, the reaction was kept at 315 °C for an additional 10 min, followed by rapid cooling to room temperature. Purification was performed following the same protocol as for the core nanoparticles, and the core-shell particles were stored in cyclohexane at 6 °C. Thicker shell variants were synthesized using the same procedure, adjusting the amount of cubic shell precursor accordingly.

1.3.3 *Ligand Exchange for Surface Modification of Nanoparticles*

For dispersion of the UCNPs in DMF or 2-cyclohexenone, surface ligands were removed from the nanoparticles using a previously established ligand exchange method.[3] For this process, 2 mL of DMF was heated to 50 °C for 5 min, followed by the dropwise addition of 1 mL of UCNPs (25 mg · mL^{-1}) in cyclohexane. The dispersion was stirred at 50 °C for 10 min, after which 50 mg of $NOBF_4$ was added. The reaction was continued under vigorous stirring at 75 °C for at least 30 min, until the mixture became clear, indicating successful ligand exchange. For purification, the nanoparticles were precipitated with an excess of chloroform and centrifuged at 1,500 *g* for 15 min. The supernatant was discarded, and the nanoparticles redispersed in the desired medium for further use.

1.4 *Calculation of Nanoparticle Concentrations*

The number of nanoparticles per mL was estimated as follows:

The theoretical volume of one nanoparticle (V_{UCNP}) was calculated according to equation (1). A quasi-spherical morphology was assumed for hexagonal $NaLnF_4$ nanoparticles <35 nm. The theoretical mass of one nanoparticle m_{UCNP} was calculated according to equation (5):

$$m_{UCNP} = \frac{V_{UCNP}}{\rho_{eff}} \tag{5}$$

Whereas the effective density ρ_{eff} of the nanoparticles is the sum of the theoretical densities ρ_{Ln} for $NaYF_4$, $NaYbF_4$ and $NaTmF_4$ (taken from ICDD PDF #00-016-0334, #00-027-1427 and #00-027-0814, respectively) times their molar doping concentrations ($c_{mol\%,\,Ln}$) determined by ICP-OES (eq. (6)).

$$\rho_{eff} = \sum c_{mol\%,\,Ln} \cdot \rho_{Ln} = c_{mol\%,\,Y} \cdot \rho_{Y} + c_{mol\%,\,Yb} \cdot \rho_{Yb} + c_{mol\%,\,Tm} \cdot \rho_{Tm} \tag{6}$$

The nanoparticle concentration N_{UCNPs} is determined by the ratio of the theoretical mass of one nanoparticle m_{UCNP} and the mass concentration β_{UCNPs}, determined by ICP-OES (eq. (7)).

$$N_{UCNPs} = \frac{\beta_{UCNPs}}{m_{UCNP}} \tag{7}$$

1.5 Calculation of Interionic Distances

The interionic distances d_{ionic} between Yb^{3+}-Tm^{3+} and Tm^{3+}-Tm^{3+} ions within a spherical nanoparticle were estimated using Equation (8), assuming a uniform distribution of the dopant ions and neglecting lattice distortions induced by doping:

$$d_{ionic} = \sqrt[3]{\left(\frac{3}{4\pi}\right)\left(\frac{\sqrt{3}/_{2} \cdot a^2 \cdot c}{c_{mol\%} \cdot n}\right)} \quad (8)$$

Where a and c are the lattice parameters of the hexagonal crystal structure, obtained from ICDD #00-016-0334 for $NaYF_4$ and ICDD #00-027-1427 $NaYbF_4$, n represents the number of lanthanide ions per unit cell (factor 1.5 for hexagonal crystal structures), and $c_{mol\%}$ is the doping ratio of the respective ions in mol%. For the Yb^{3+}-Tm^{3+} interionic distance, $c_{mol\%}$ was taken as the sum of the doping concentrations of Yb^{3+} and Tm^{3+} (in mol%) ensuring an accurate estimation of their spatial distribution within the nanoparticle lattice.

1.6 Laser Diode Setup for Indirect Excitation of Photosensitive Reactions

The laser diode system used for indirect excitation of photosensitive reactions consisted of a high-power 3 W, 975 nm (cw) laser diode source, coupled with a beam-shaping unit. The beam-shaping setup included two plano-convex lenses of equal focal length and a biconcave lens with an absolute focal length less than half of the plano-convex lenses. To determine the laser power density, the cross-sectional dimensions of the laser beam were estimated. A UCNP-coated paper with printed markings was placed in the laser path, and photographs were taken at 50° relative to the laser setup for different positions of lens L_2. The images were processed to extract the distortion factor, enabling an estimation of the beam width by measuring the outer dimensions of the upconversion emission, assuming a rectangular beam profile. Based on the maximum laser power and the calculated beam dimensions, power densities were derived as a function of the position of lens L_2.

1.7 Photocatalytic Reactions under NIR excitation

For photoreactions under indirect NIR excitation, UCNPs dispersed in cyclohexane, DMF, or chloroform (volume, nanoparticle concentrations, and absolute nanoparticle quantities are specified for the respective reaction) were added to the initial materials in a 2 mm × 10 mm UV quartz microcuvette, which was equipped with a miniature stirring bar. The cuvette was quickly flushed with an argon atmosphere and sealed to create an inert atmosphere. The

cuvette was placed in the focused 975 nm laser diode beam and irradiated for an extended time at room temperature while stirring. To prevent clogging due to product formation and obstruction of the laser beam, the cuvette was repositioned along the z-axis every 30 minutes. The crude reaction mixture was either characterized directly, or the UCNPs were collected by precipitation with DMSO-d_6 and centrifuged at 21,000 *g* for 30 minutes. The supernatant was collected and characterized, and the UCNPs were redispersed in chloroform. The collected UCNPs were then ready for further use as photocatalysts. Recovery was determined by ICP-OES.

To monitor the temperature over time, a vial containing UCNPs in cyclohexane at mass concentrations comparable to those utilized during the photocatalytic reactions was placed in the laser beam. In a controlled environment, a thermographic camera was utilized to systematically record the ambient temperature, the lowest recorded temperature, and the highest recorded temperature within a defined area over the course of 120 minutes.

1.7.1 Dimerization of 2-Cyclohexenone

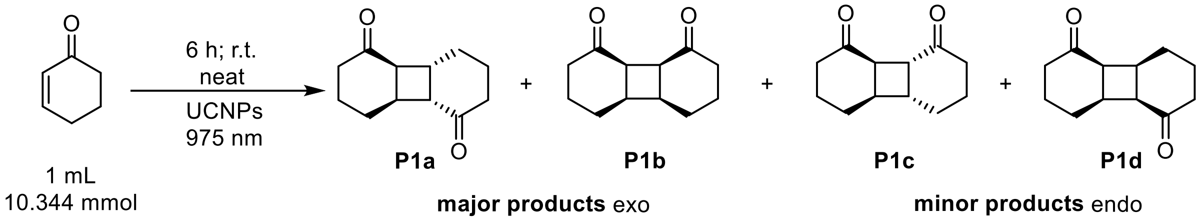

Neat conditions.

Calibration performed by GC.

External standard used 1,4-bis-trifluoromethylbenzene.

The yield of the targeted product: 0.66% - 0.034 mmol;

Quantity of UCNPs – 2.27×10^{14}. Number of UCNPs – 2.27×10^{14} mL^{-1}.

$$\boldsymbol{TON} = 0.000034\frac{N_A}{2.27 \times 10^{14}} = \mathbf{90167}$$

$$\boldsymbol{TOF} = \frac{90167}{21600\ \mathrm{s}} = \mathbf{4.17\ s^{-1}}$$

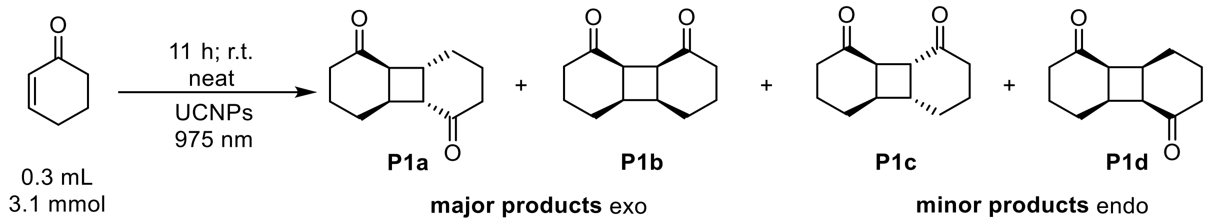

Neat conditions.

Calibration performed by GC.

External standard used 1,4-bis-trifluoromethylbenzene.

Percentage of starting material left after reaction: 92.8%

The yield of the targeted product: 3.01% - 0.096 mmol;

Selectivity – exo:endo = 6.4:1

Selectivity of diastereomers – 8.4:6.2:1.3:1

Quantity of UCNPs – $1.07{\times}10^{15}$. Number of UCNPs – $3.2{\times}10^{14}$ mL^{-1}.

$$\boldsymbol{TON} = 0.000096\frac{N_A}{3.2 \times 10^{14}} = \mathbf{180600}$$

$$\boldsymbol{TOF} = \frac{180600}{39600\ \mathrm{s}} = \mathbf{4.56\ s^{-1}}$$

1.7.2 Dimerization of 4-(2,5-Dioxo-2,5-Dihydro-1H-Pyrrol-1-yl)Butanoic Acid

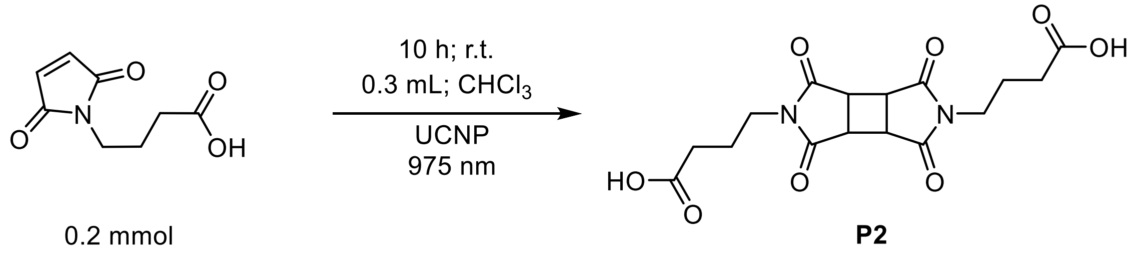

Solvent: Chloroform.

Yields were derived from ^{1}H NMR data.

External standard used 1,4-bis-trifluoromethylbenzene.

Percentage of starting material left after reaction: 91%

The yield of the targeted product (single diastereomer): 4.44% - 0.00888 mmol;

Quantity of UCNPs – $1.52{\times}10^{14}$. Number of UCNPs – $5.07{\times}10^{14}$ mL^{-1}.

$$\boldsymbol{TON} = 0.00000888\frac{N_A}{1.52 \times 10^{14}} = \mathbf{35170}$$

$$\boldsymbol{TOF} = \frac{35170}{36000\ \mathrm{s}} = \mathbf{0.977\ s^{-1}}$$

1.7.3 2+2 Photocycloaddition between 3,5,5-Trimethylcyclohex-2-En-1-One and 3,4-Dihydro-2H-Pyran

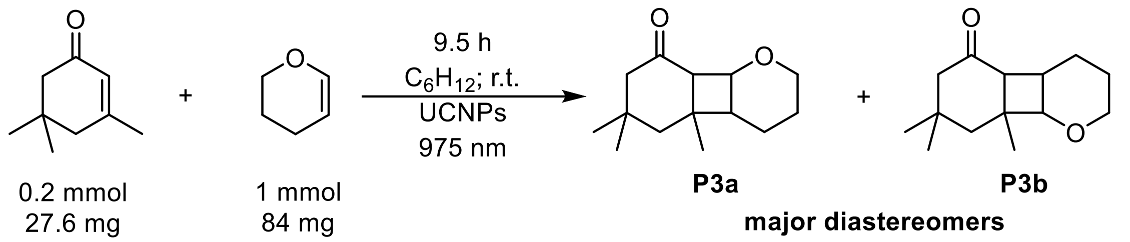

Solvent: Cyclohexane.

Calibration performed by GC.

External standard used 1,4-bis-trifluoromethylbenzene.

Percentage of starting material left after reaction: 67%

The yield of the targeted products: 30% (0.06 mmol)

Quantity of UCNPs - 1.24×10^{14}.

$$\boldsymbol{TON} = 0.00006 \frac{N_A}{1.24 \times 10^{14}} = \mathbf{291291}$$

$$\boldsymbol{TOF} = \frac{291291}{34200\ \mathrm{s}} = \mathbf{8.52\ s^{-1}}$$

1.7.4 Paternò–Büchi Reaction Between Cyclohexene and Benzaldehyde

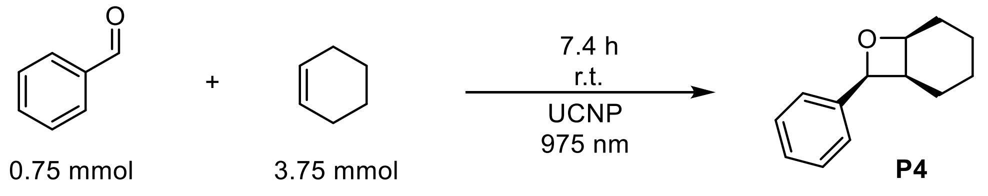

Solvent: dimethyl formamide.

Single diastereomer.

(Reaction time – 7 h and 25 min).

Calibration has been done on GC.

External standard used 1,4-bis-trifluoromethylbenzene.

Percentage of starting material left after reaction: 94.6%

The yield of the targeted product (single diastereomer): 2.66% (0.02 mmol)

Quantity of UCNPs - $9.86{\times}10^{13}$.

$$\boldsymbol{TON} = 0.00002\frac{N_A}{9.86 \times 10^{13}} = \mathbf{122110}$$

$$\boldsymbol{TOF} = \frac{122110}{26700\ \mathrm{s}} = \mathbf{4.57\ s^{-1}}$$

1.8 Preparation of Substrates under Direct UVA Irradiation

1.8.1 P1(a-d)

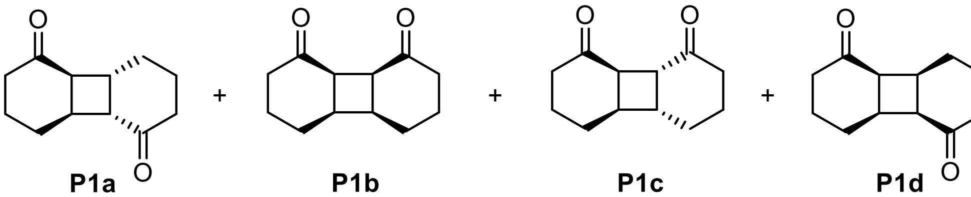

P1a **P1b** **P1c** **P1d**

major products exo **minor products** endo

Preparation: Under the inert atmosphere, a 5 mL crimp-capped vial was charged with a stirring bar, and cyclohexanone 480 mg (5 mmol). The substrate was stirred and irradiated by 365 nm, (~3W) LED for 2 h at room temperature (until it was completely consumed). The reaction yielded nearly a quantitative diastereomeric mixture of the [2+2] photocycloaddition products as transparent-yellowish highly viscous liquid.

Exo:endo selectivity = 4.48:1

Ratio of diastereomers – 5.83:4.03:1.2:1

^{1}H NMR (400 MHz, $CDCl_3$) δ = 1.39-1.51 (1.10H), 1.55-1.70 (2.17H), 1.70-1.99 (4.89H), 2.01-2.34 (4.68H), 2.50-2.64 (0.81H), 2.64-2.86 (1.36H), 2.86-3.27 (1.81H) ppm.

(*) ^{1}H NMR (400 MHz, $CDCl_3$) reported for diastereomeric mixture.

^{13}C{^{1}H} NMR (101 MHz, $CDCl_3$) **P1a** δ = 21.3, 26.8, 38.2, 40, 47.4 213 ppm. **P1b** δ = 22.6, 24.9, 40, 41, 47.5, 213.2 ppm.

HRMS: *m/z* for $[C_{12}H_{16}O_2]^+$ [M $^+$] calcd: 192.1145, found: 192.1149.

Assignment and interpretation of spectral data was based on previously reported literature.[4,5]

1.8.2 4,4'-(1,3,4,6-Tetraoxooctahydrocyclobuta[1,2-c:3,4-c']Dipyrrole-2,5-diyl)Dibutyric Acid (P2)

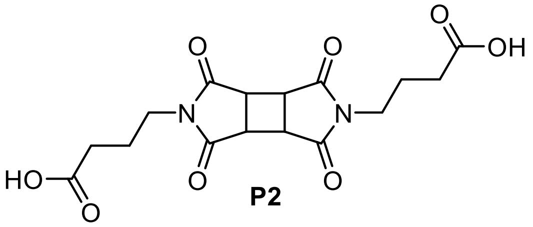

P2

Preparation: Under the inert atmosphere, a 5 mL crimp-capped vial was charged with a stirring bar, and 4-(2,5-dioxo-2,5-dihydro-1H-pyrrol-1-yl)butanoic acid (0.5 mmol) and 1 mL of chloroform. The reaction mixture was stirred vigorously and irradiated *via* 365 nm, (~3W) LED for 2 h at room temperature (until the starting material was completely consumed). The reaction yielded nearly quantitative 4,4'-(1,3,4,6-tetraoxooctahydrocyclobuta[1,2-c:3,4-c']dipyrrole-2,5-diyl)dibutyric acid as a single diastereomer. White amorphous solid.

^{1}H NMR (400 MHz, DMSO) δ = 1.99 (m, 4H), 2.49 (t, *J* = 7.24 Hz, 4H), 3.57 (s, 4H), 3.71 (t, *J* = 6.82 Hz, 4H), 12.27 (br. s, 1H) ppm.

^{13}C{^{1}H} NMR (101 MHz, DMSO) δ = 22.8, 31.5, 38.7, 41.4, 174.4, 176.6 ppm.

HRMS: *m/z* for ([$C_{16}H_{18}N_2O_8$]+H $^+$) [(M +H)$^+$] calcd: 367.1136, found: 367.1126.

The compound has been previously reported without spectral characterization.[6]

1.8.3 P3(a-b)

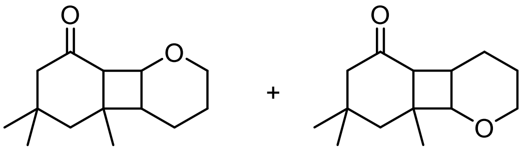

P3a **P3b**

Preparation: Under the inert atmosphere, a 5 mL crimp-capped vial was charged with a stirring bar, 3,5,5-trimethylcyclohex-2-en-1-one (0.5 mmol) and 3,4-dihydro-2H-pyran (0.5 mmol). The reaction mixture was stirred vigorously and irradiated by 365 nm, (~3W) LED for 1 h at room temperature (until the starting materials were completely consumed). The reaction yielded nearly quantitative a diastereomeric mixture of 2+2 photocycloaddition products.

Two major diastereomers formed in a diastereomeric ratio: 58:42

Spectral characterization of one isolated diastereomer, (identified as P3b).

^{1}**H NMR** (400 MHz, $CDCl_3$) δ = 0.93 (s, 3H), 1.06 (s, 3H), 1.16 (s, 3H), 1.40-1.47 (m, 1H), 1.50-1.63 (m, 3H), 1.67-1.83 (m, 2H), 2.01 (m, 1H), 2.43 (d, *J* = 14.78 Hz, 1H), 2.58 (m, 1H), 2.75 (d, *J* = 10.15 Hz, 1H), 3.19 (td, ^{3}J = 11.64 Hz, ^{4}J = 1.68 Hz, 1H), 3.67 (d, *J* = 4.84 Hz, 1H) 3.86 (m, 1H) ppm.
^{13}**C{^{1}H} NMR** (101 MHz, $CDCl_3$) δ = 21.2, 22.2, 22.8, 27.6, 32.4, 33.3, 36.3, 42.1, 45.4, 50.8, 52.4, 64.4, 77.9, 213.1 ppm
Spectral characterization of the second diastereomer (identified as P3a) based on the analysis of the crude spectrum.
^{1}**H NMR** (400 MHz, $CDCl_3$) *Incomplete only clearly identifiable peaks are reported δ = 0.89 (s, 3H), 1.08 (s, 3H), 1.31 (s, 3H),..., 4.02 (m, 1H) ppm.
^{13}**C{^{1}H} NMR** (101 MHz, $CDCl_3$) δ = 27.1, 27.7, 28.9, 29.4, 32.2, 36.7, 38.9, 44.5, 50.5, 51, 53.4, 69.3, 85.6, 211.2 ppm.
HRMS: *m/z* for ($[C_{14}H_{22}O_2]$+H $^{+}$) $[(M+H)^{+}]$ calcd: 223.1693, found: 223.1697.

1.8.4 Endo-8-Phenyl-7-Oxabicyclo[4.2.0]Octane (P4)

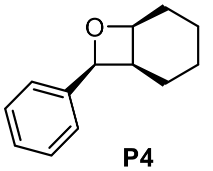

P4

Preparation: Under the inert atmosphere, a 5 mL crimp-capped vial was charged with a stirring bar, benzaldehyde 212 mg (2 mmol), and cyclohexene 822 mg (10 mmol). The mixture was stirred and irradiated *via* 365 nm LED for 2 h (until benzaldehyde was completely consumed). An excess of olefin was removed under reduced pressure, and the crude mixture was purified on column (ethyl acetate : petrol ether = 1:9)
Isolated yield: 37% (139 mg white solid).
^{1}**H NMR** (400 MHz, $CDCl_3$) δ = 0.98-1.01 (m, 1H), 1.21-1.33 (m, 2H), 1.44-1.56 (m, 5H), 1.90 (m, 1H), 2.96 (m, 1H), 4.99 (m, 1H), 5.87 (d, *J* = 6.32 Hz, 1H), 7.20-7.28 (m, 3H). 7.29-7.39 (m, 2H) ppm.
^{13}**C{^{1}H} NMR** (101 MHz, $CDCl_3$) δ = 20.1, 21.3, 21.5, 29, 37.5, 75.7, 81.6, 125.3, 126.8, 128.1, 140.2 ppm.
HRMS: *m/z* for $[C_{13}H_{16}O]^{+}$ $[M^{+}]$ calcd: 188.1196, found: 188.1195.
Spectral data matches those reported in the literature.[7]

2 Supporting Data and Figures

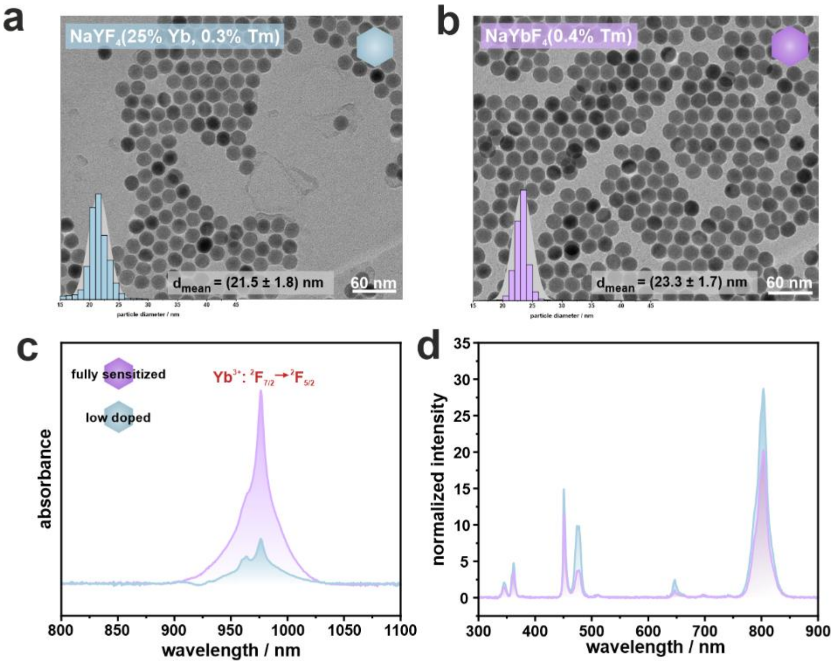

Figure S1: Characterization of low doped ($NaYF_4$(25% Yb, 0.3% Tm)) and fully sensitized ($NaYbF_4$(0.4% Tm)) core nanoparticles. TEM micrographs of (a) $NaYF_4$(25% Yb, 0.3% Tm) (diameter (21.5 ±1.8) nm) and (b) $NaYbF_4$(0.4% Tm) (diameter (23.3 ±1.7) nm) nanoparticles with corresponding size distribution histogram. (c) Absorbance spectra of low doped and fully sensitized nanoparticles. The spectra were normalized to the absorbance per particle. (d) Luminescence spectra of fully sensitized and low doped nanoparticles in cyclohexane under 980 nm (cw) excitation with a power density of 150 W cm^{-2}. The spectra were normalized to the particle concentration.

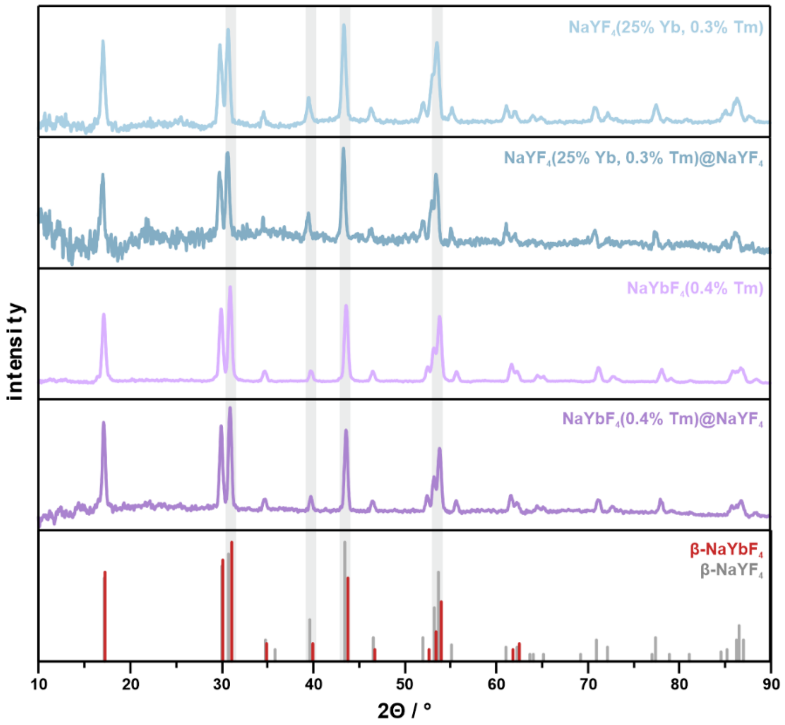

Figure S2: Powder X-Ray diffraction of core and core-shell nanoparticles of low doped and fully sensitized particles. Reference diffraction pattern of β-$NaYbF_4$ (red, ICDD #00-027-1427) and β-$NaYF_4$ (gray, ICDD #00-016-0334). The light gray bars serve as guidance for the eye.

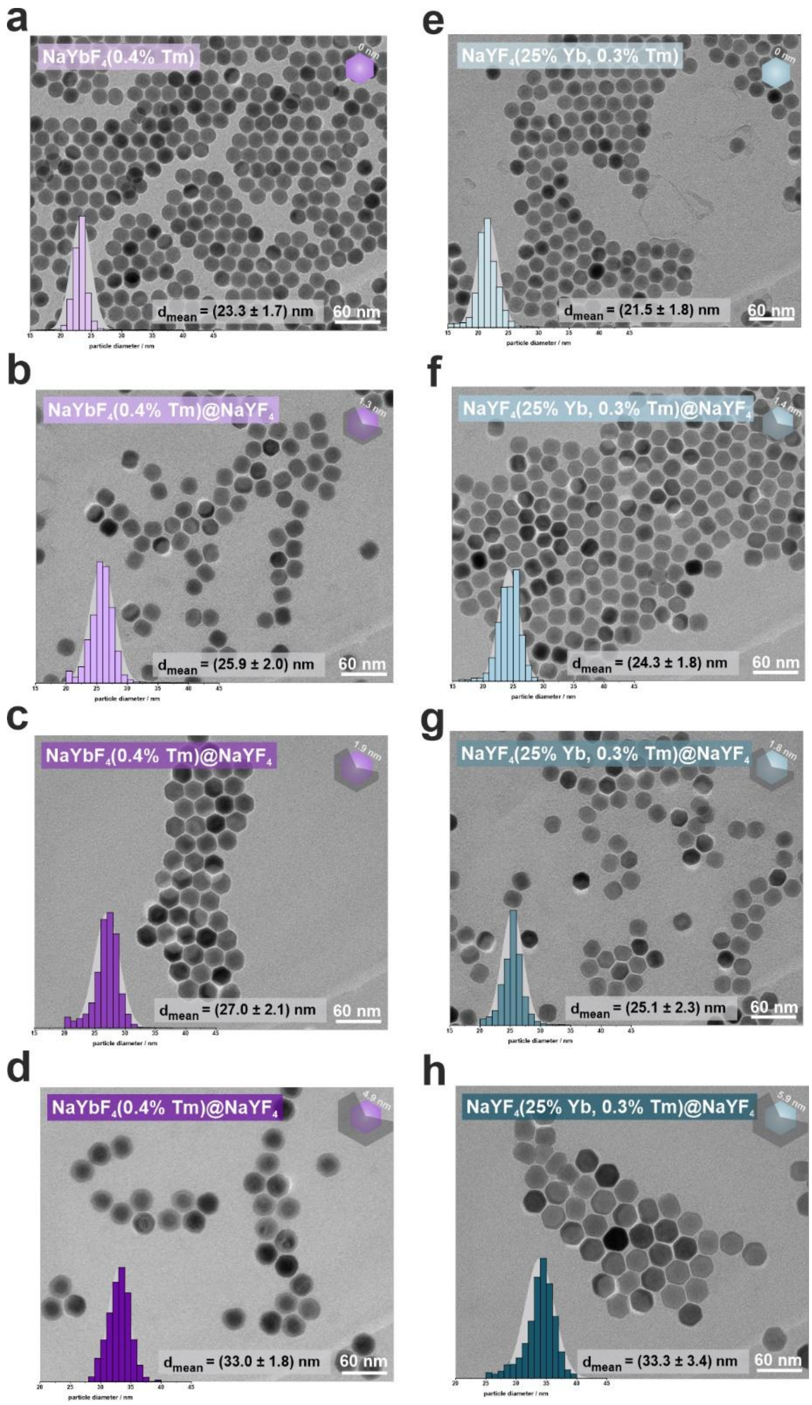

Figure S3: TEM micrographs of fully sensitized $NaYbF_4$(0.4% Tm) and low doped $NaYF_4$(25% Yb, 0.3% Tm) core and core-shell nanoparticles with varying inert shell thicknesses. (**a-d**) TEM micrographs of fully sensitized $NaYbF_4$(0.4% Tm) core and core-shell nanoparticles with corresponding size distribution histogram and respective particle diameter. (**e-h**) TEM micrographs of low doped $NaYF_4$(25% Yb, 0.3% Tm) core and core-shell nanoparticles with size distribution histogram and respective particle diameter. The inset in the TEM micrographs reflects the respective shell thickness.

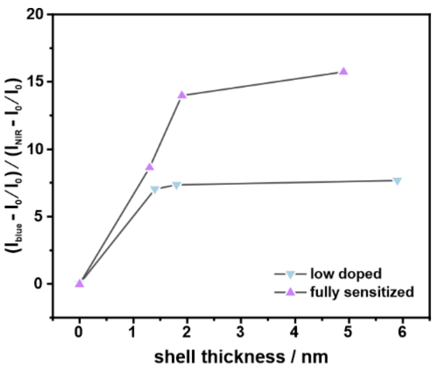

Figure S4: Relative change in blue-to-NIR intensity ratio (integration range 435 – 491 nm for blue emission and 765 – 851 nm for NIR) normalized to the emission intensity of the respective emission band of the core particles as a function of the optically inert shell thickness of both low doped and fully sensitized nanoparticles. The luminescence spectra were recorded in cyclohexane under 980 nm (cw) excitation with a power density of 150 W cm^{-2} and normalized to the particle concentration.

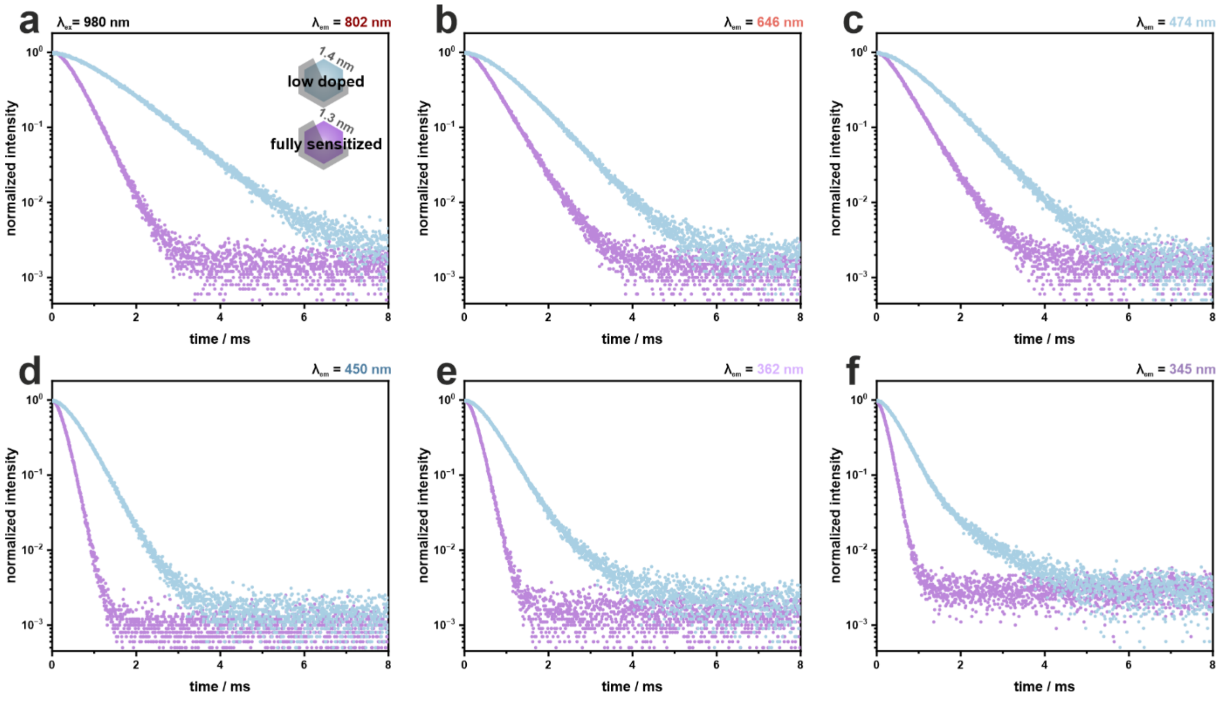

Figure S5: Luminescence decay curves of Tm^{3+} for low doped $NaYF_4$(25% Yb, 0.3% Tm)@$NaYF_4$ and fully sensitized $NaYbF_4$(0.4% Tm)@$NaYF_4$ nanoparticles with a 1.5 nm thin shell. The decays were recorded under 980 nm (pulsed) excitation in cyclohexane under the same excitation power density and a pulse width of 150 µs.

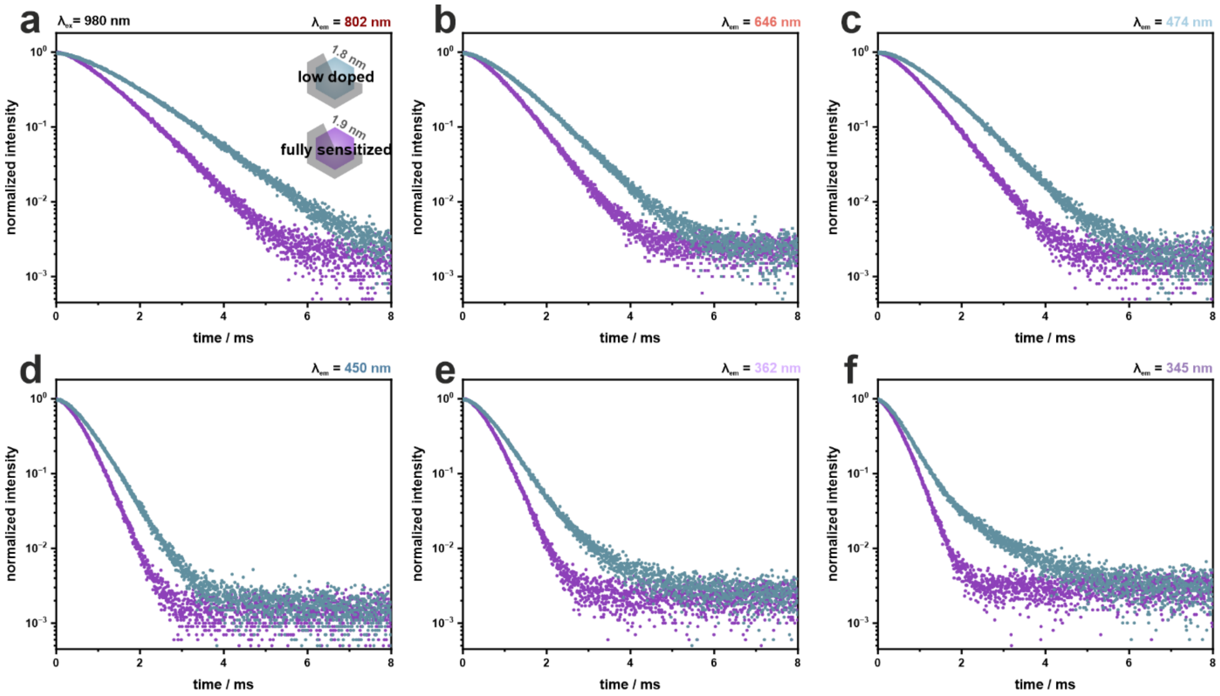

Figure S6: Luminescence decay curves of Tm^{3+} for low doped $NaYF_4$(25% Yb, 0.3% Tm)@$NaYF_4$ and fully sensitized $NaYbF_4$(0.4% Tm)@$NaYF_4$ nanoparticles with a 2 nm thin shell. The decays were recorded under 980 nm (pulsed) excitation in cyclohexane under the same excitation power density and a pulse width of 150 µs.

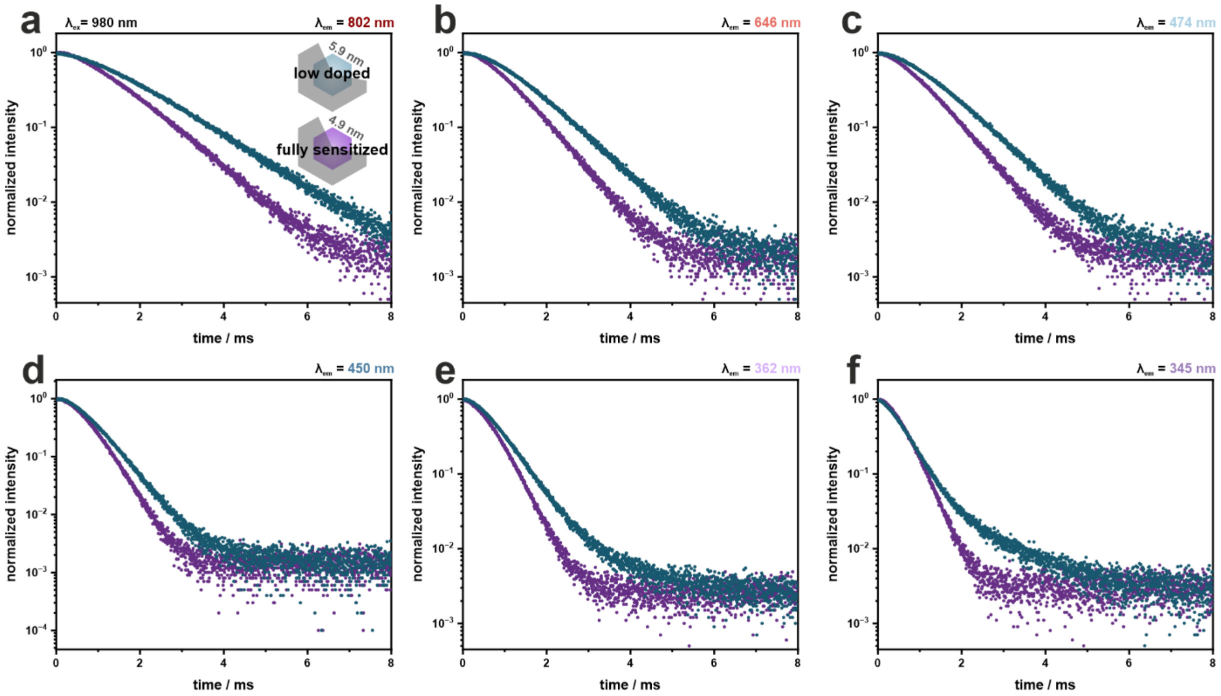

Figure S7: Luminescence decay curves of Tm^{3+} for low doped $NaYF_4$(25% Yb, 0.3% Tm)@$NaYF_4$ and fully sensitized $NaYbF_4$(0.4% Tm)@$NaYF_4$ nanoparticles with a 5 nm thick shell. The decays were recorded under 980 nm (pulsed) excitation in cyclohexane under the same excitation power density and a pulse width of 150 µs.

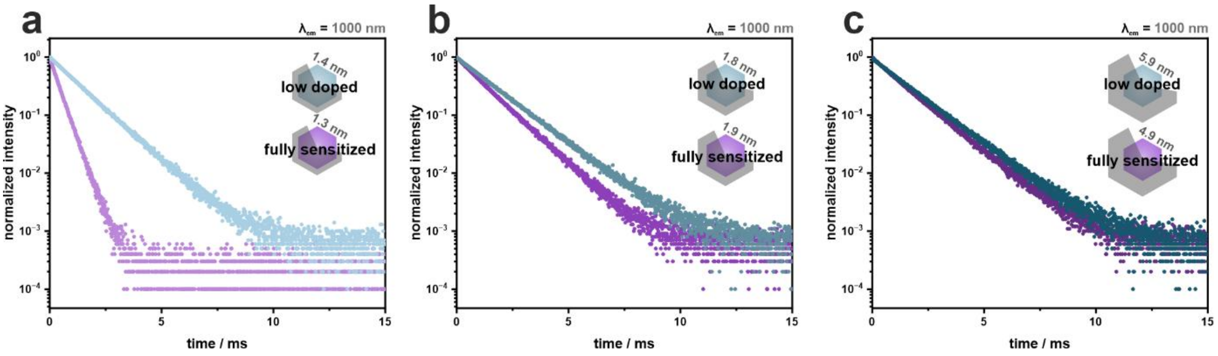

Figure S8: Luminescence decay curves of Yb^{3+} for low doped $NaYF_4$(25% Yb, 0.3% Tm)@$NaYF_4$ and fully sensitized $NaYbF_4$(0.4% Tm)@$NaYF_4$ nanoparticles with a 1.5 nm (a), 2 nm (b) and 5 nm (c) thick shell. The decays were recorded under 980 nm (pulsed) excitation in cyclohexane under the same excitation power density and a pulse width of 150 µs.

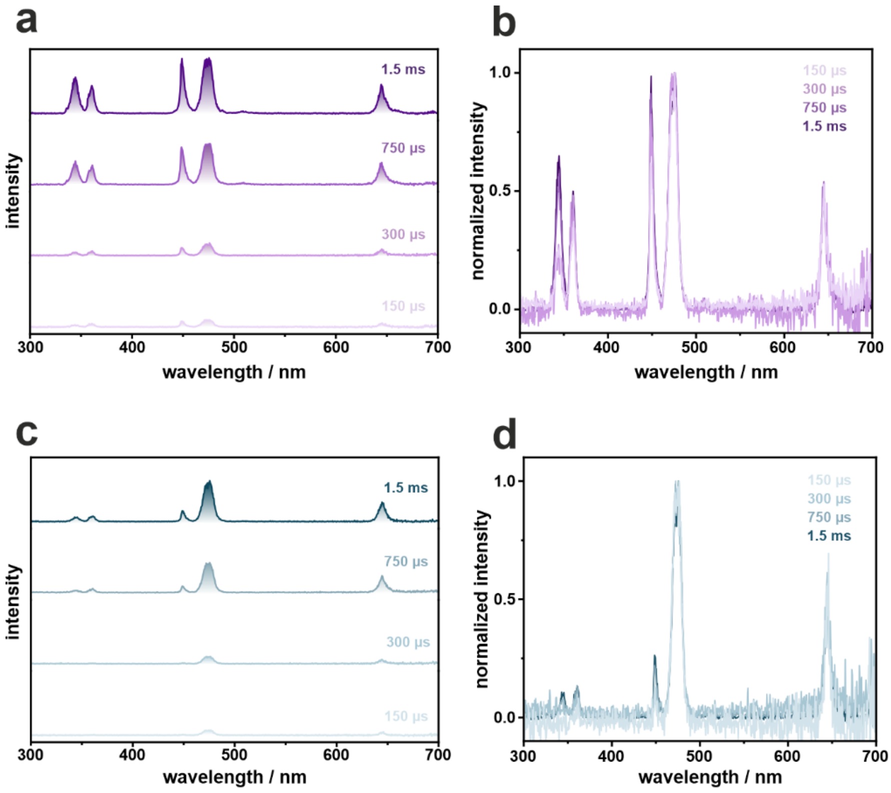

Figure S9: Impact of the pulse width on the luminescence properties of the nanoparticles. Luminescence spectra of fully sensitized $NaYbF_4$(0.4% Tm)@$NaYF_4$ (a, b) and low doped $NaYF_4$(25% Yb, 0.3% Tm)@$NaYF_4$ (c, d) nanoparticles with a 5 nm thick shell under pulsed 980 nm excitation with varying pulse widths and constant duty cycle (3.75%). The luminescence spectra were recorded in cyclohexane under 980 nm (cw) excitation and normalized to the maximum intensity of the 474 nm emission (b, d).

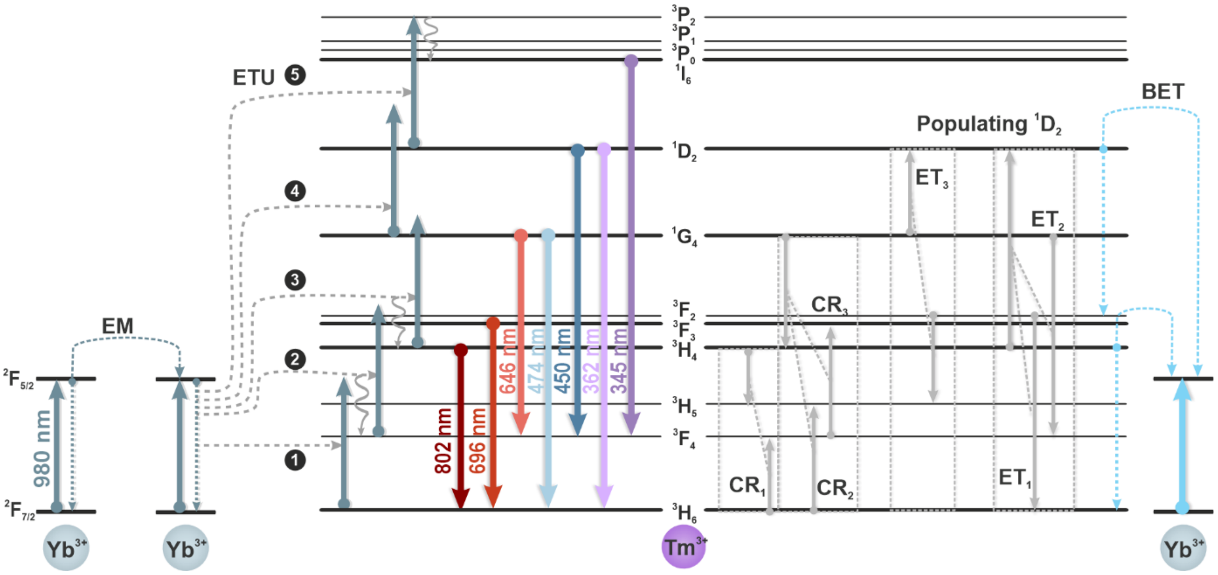

Figure S10: Energy level scheme of Yb^{3+}, Tm^{3+} co-doped nanoparticles taking different possible energy transfer mechanisms into account. EM: energy migration, ETU: energy transfer upconversion, CR: cross-relaxation, ET: energy transfer and BET: back-energy transfer.

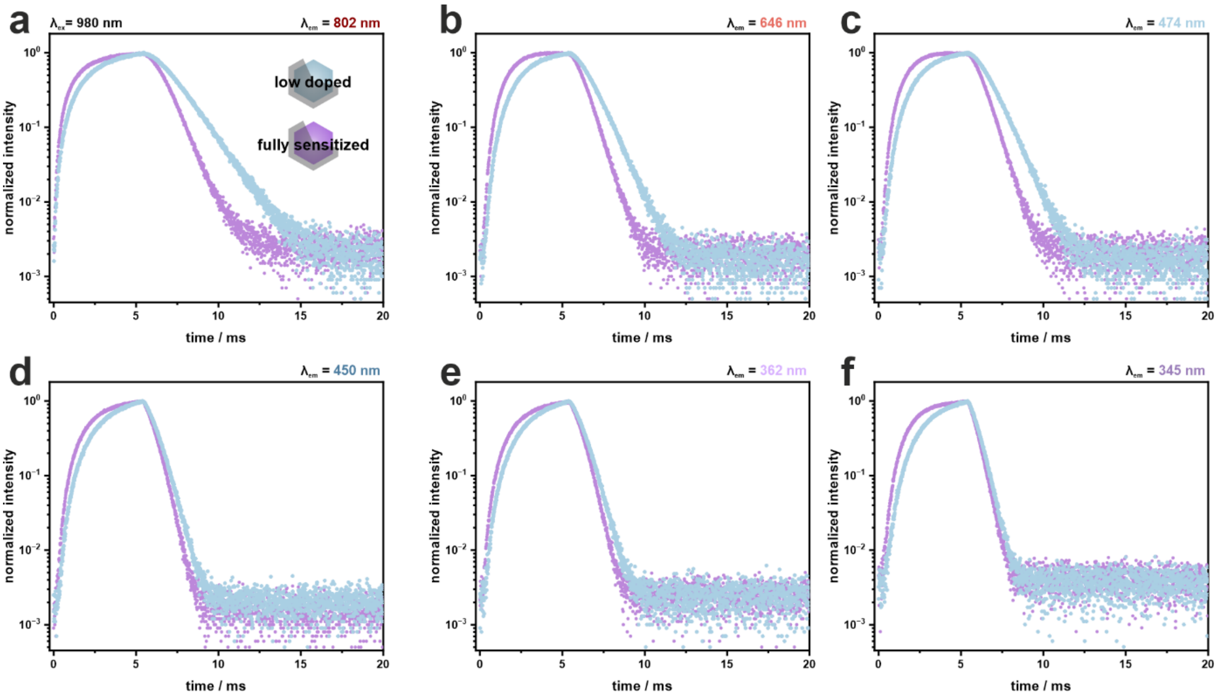

Figure S11: Luminescence rise and decay curves for low doped $NaYF_4$(25% Yb, 0.3% Tm)@$NaYF_4$ and fully sensitized $NaYbF_4$(0.4% Tm)@$NaYF_4$ nanoparticles with a 5 nm thick shell under 980 nm (pulsed) excitation in cyclohexane under the same excitation power density and a pulse width of 5 ms.

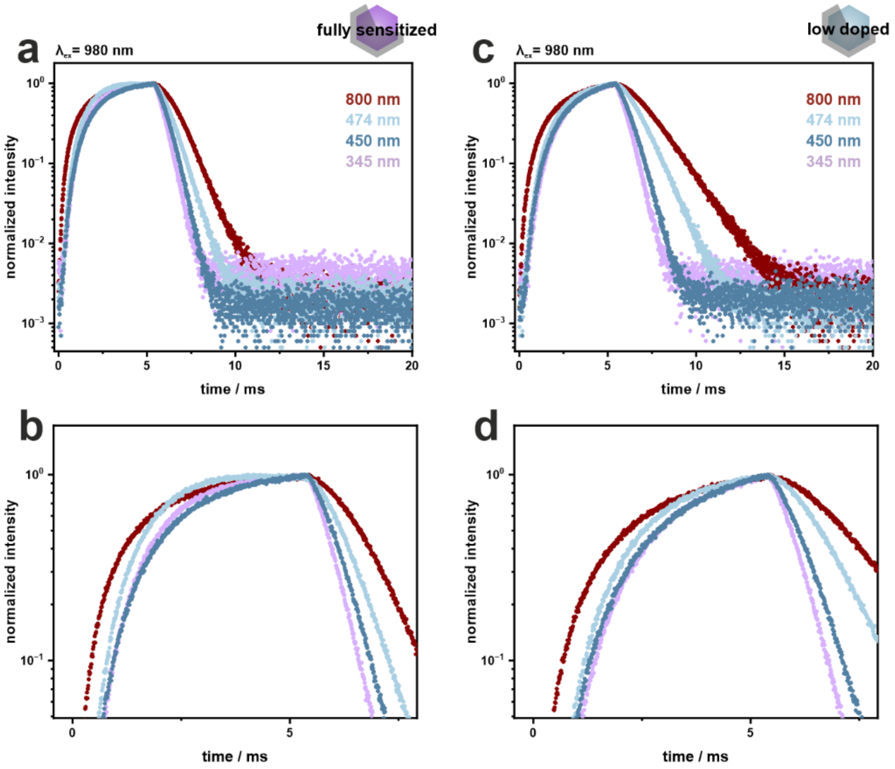

Figure S12: Comparison of the luminescence rise and decay curves for low doped $NaYF_4$(25% Yb, 0.3% Tm)@$NaYF_4$ and fully sensitized $NaYbF_4$(0.4% Tm)@$NaYF_4$ nanoparticles with a 5 nm thick shell at different emission wavelengths. Luminescence rise and decay curves for fully sensitized (**a**) and low doped (**c**) nanoparticles. (**b, d**) Zoom in on the regime of the rise curve. All curves were recorded in cyclohexane under 980 nm (pulsed) excitation under the same excitation power density and a pulse width of 5 ms. For better visualization, only the brightest emission bands of the respective energy levels were plotted.

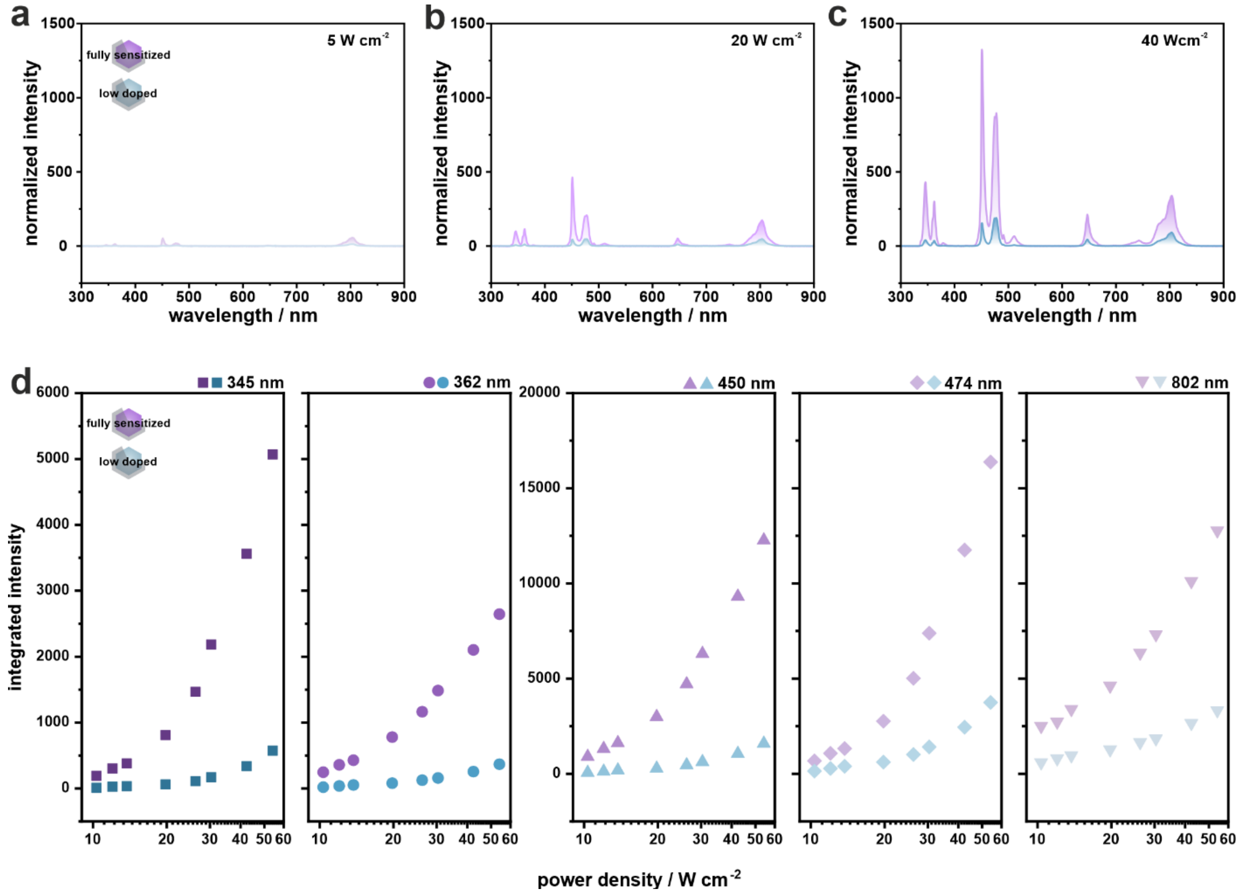

Figure S13: Power density dependency of the luminescence of low doped $NaYF_4$(25% Yb, 0.3% Tm)@$NaYF_4$ and fully sensitized $NaYbF_4$(0.4% Tm)@$NaYF_4$ core-shell nanoparticles. Luminescence spectra of fully sensitized and low doped nanoparticles in cyclohexane under 980 nm (cw) excitation with power densities of 5 W cm^{-2} (**a**), 20 W cm^{-2} (**b**), and 40 W cm^{-2} (**c**). All spectra were normalized to the particle concentration. (**d**) Luminescence intensity as a function of excitation power density for the 345 nm, 362 nm, 450 nm, 474 nm, and 802 nm emissions, for fully sensitized and low doped particles, respectively, under 980 nm (cw) excitation in cyclohexane.

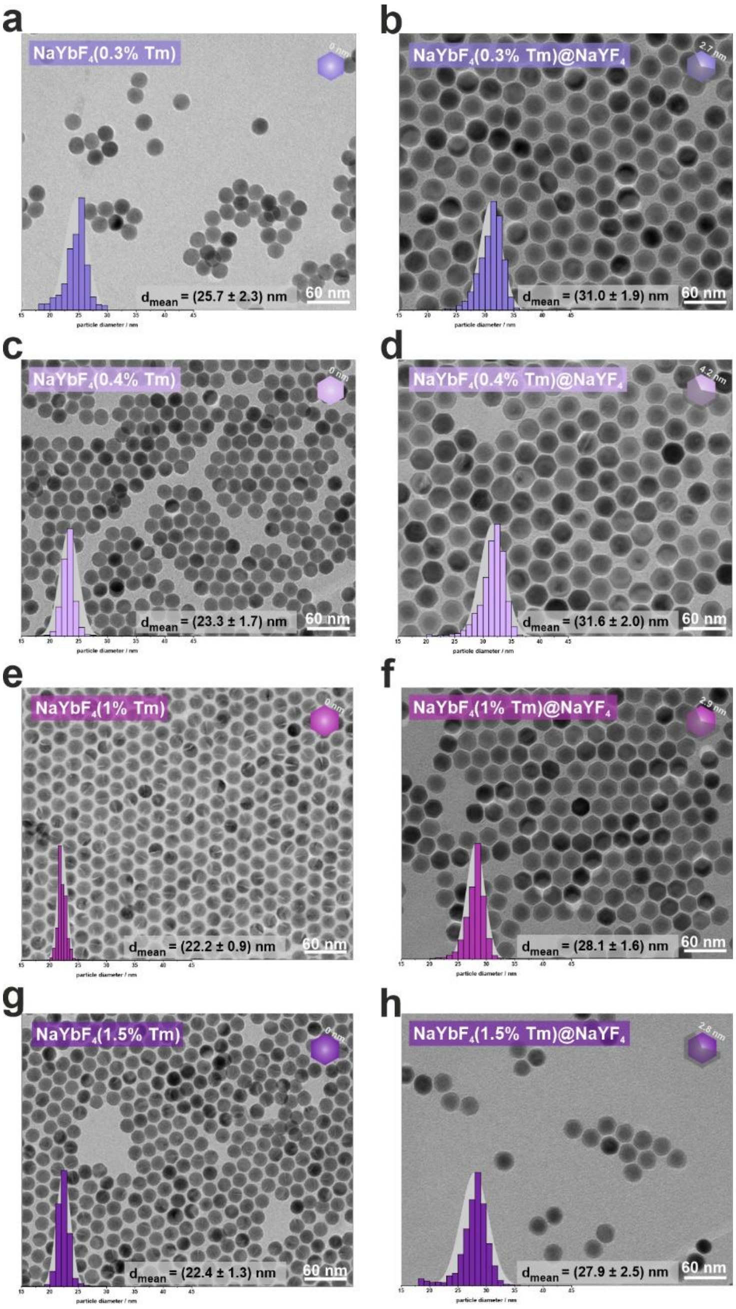

Figure S14: TEM micrographs with corresponding size distribution histograms and respective particle diameter of fully sensitized $NaYbF_4$(x% Tm) core and core-shell nanoparticles with varying Tm^{3+} concentrations (x = 0.3, 0.4, 1, 1.5). The inset in the TEM micrographs reflects the respective shell thickness.

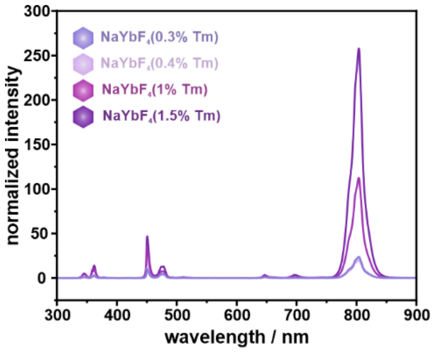

Figure S15: Luminescence spectra of $NaYbF_4$(x% Tm^{3+}) core nanoparticles with varying Tm^{3+} concentrations (x(Tm^{3+}) = 0.3, 0.4, 1, 1.5). The spectra were recorded in cyclohexane under 980 nm (cw) excitation with a power density of 150 W cm^{-2}. The spectra were normalized to the particle concentration.

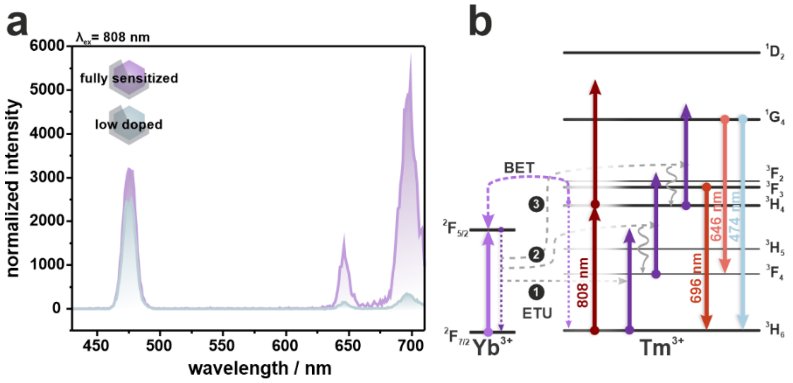

Figure S16: Direct excitation of Tm^{3+}. (**a**) Luminescence spectra of low doped $NaYF_4$(25% Yb, 0.3% Tm)@$NaYF_4$ and fully sensitized $NaYbF_4$(0.4% Tm)@$NaYF_4$ nanoparticles in cyclohexane under 808 nm (cw) excitation. The spectra were normalized to the particle concentration. (**b**) Energy level scheme of Yb^{3+}, Tm^{3+} co-doped nanoparticles under 808 nm excitation. BET: back-energy transfer and ETU: energy transfer upconversion.

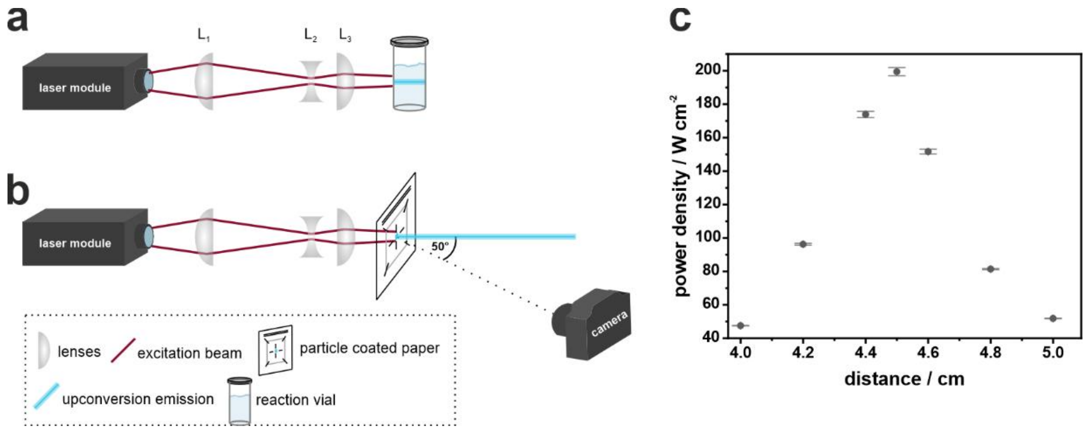

Figure S17: Home-built laser setup for the photosensitive reactions under indirect NIR excitation. (a) Scheme of the laser setup including a 3 W, 975 nm (cw) laser module and different plano-convex (L_1 and L_3) and biconcave (L_2) lenses for beam shaping of the incident laser beam. (b) Scheme of the determination of the laser beam cross-section in the setup. (c) Determination of the maximum power density of the 3 W, 975 nm, cw laser module dependent on the x-position of from L_1.

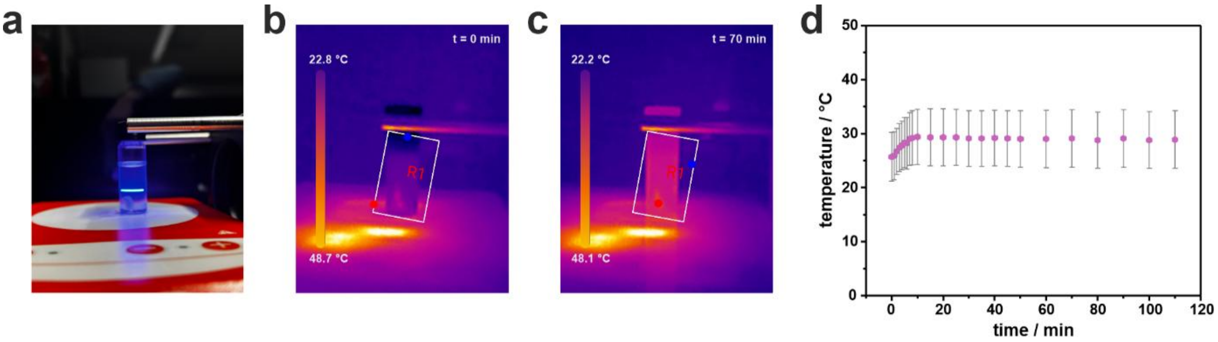

Figure S18: Monitoring of excitation induced heating effects in a UCNP dispersion in cyclohexane (β≈40 mg mL^{-1}). (**a**) Photograph of fully sensitized UCNPs in cyclohexane under 3 W, 980 nm, cw excitation. Thermographic images of UCNPs in cyclohexane at t = 0 min (**b**) and t = 70 min (**c**). The inset indicates the temperature scale. (**d**) Temporal development of the temperature of the UCNPs in cyclohexane under 980 nm excitation.

Table S2: Comparison of different upconversion processes for the generation of UV to trigger photoreactions. TTA-UC: triplet-triplet upconversion and TPA: two-photon absorption.

UC Process for UV-generation in homogenous or micro heterogenous media	Excitation wavelength	Emission wavelength	Inherent energy loss**	Required excitation light intensity	Light source requirements	Reference
TTA-UC	a. 445 nm<br>b. 445 nm	a. 372 nm<br>b. 299 nm	a. 40%<br>b. 25.6%	a.*** $2.3x10^{-3}$ W cm^{-2}<br>b. $4.71x10^{-1}$ W cm^{-2}	a. LED/solar<br>b. LED	a. [8]<br>b. [9]
Second-harmonic nanoparticles	790 nm	395 nm	0%	$4.3x10^{11}$ W cm^{-2}	pulsed laser	[10]
TPA	To the best of our knowledge, no example of a UV emitter TPA dye has been reported that leads to photochemical transformations.					
Our method	980 nm	345 nm	43%	$1.5x10^{2}$ W cm^{-2}	cw laser	

** Inherent energy loss refers to the energy difference between the combined input photons and the single upconverted photon

*** Threshold excitation intensity

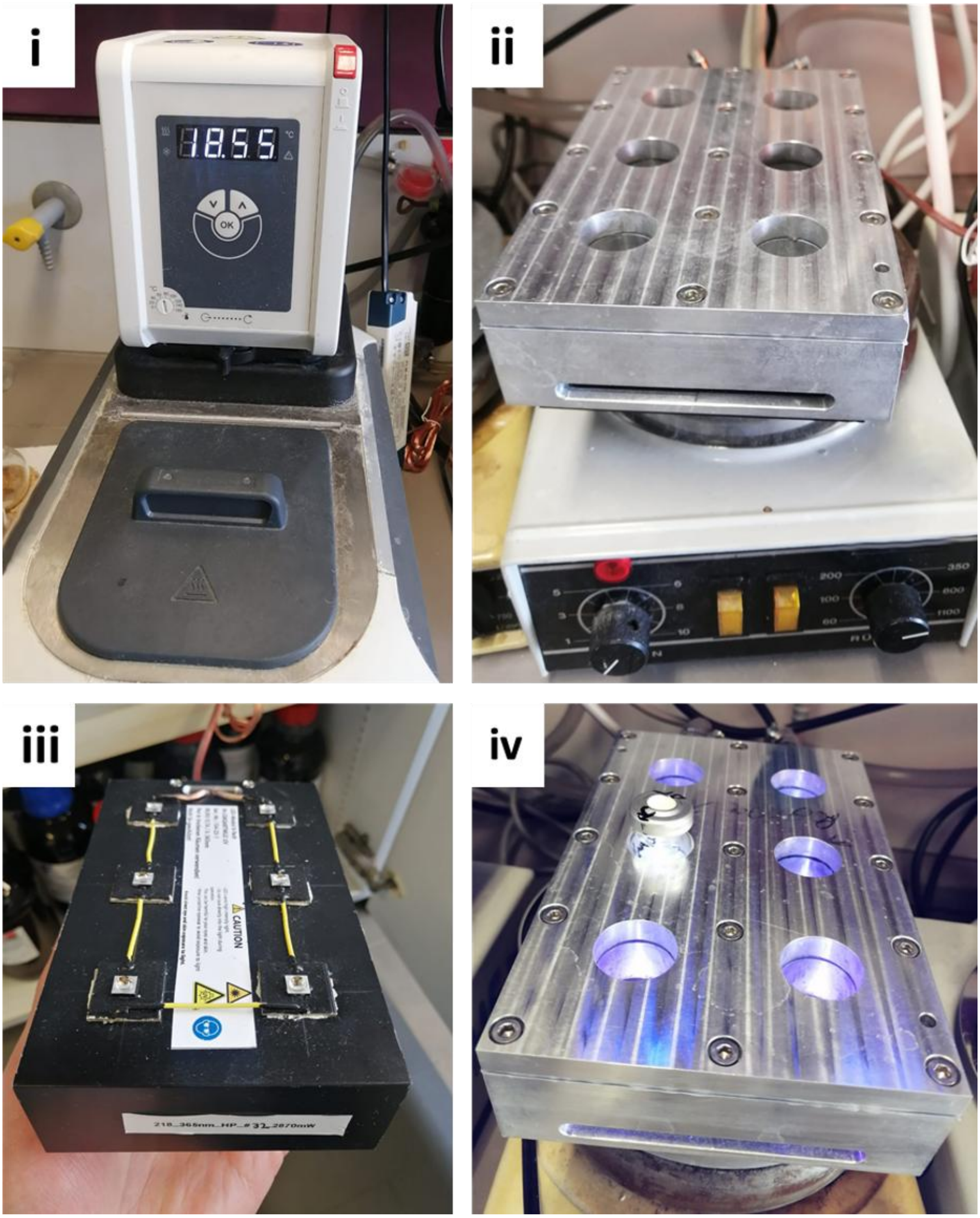

Figure S 19: Overview of the set-up for the control photoreactions, carried out under 365 nm excitation. (i) Temperature controller. (ii) Stirring plate with an aluminum cooling block on top. (iii) 365 nm LEDs with an optical power of 2870 mW. (iv) Reaction conducted in a 5 mL crimped-cap vial placed on the aluminum cooling block under irradiation.

3 NMR Spectra

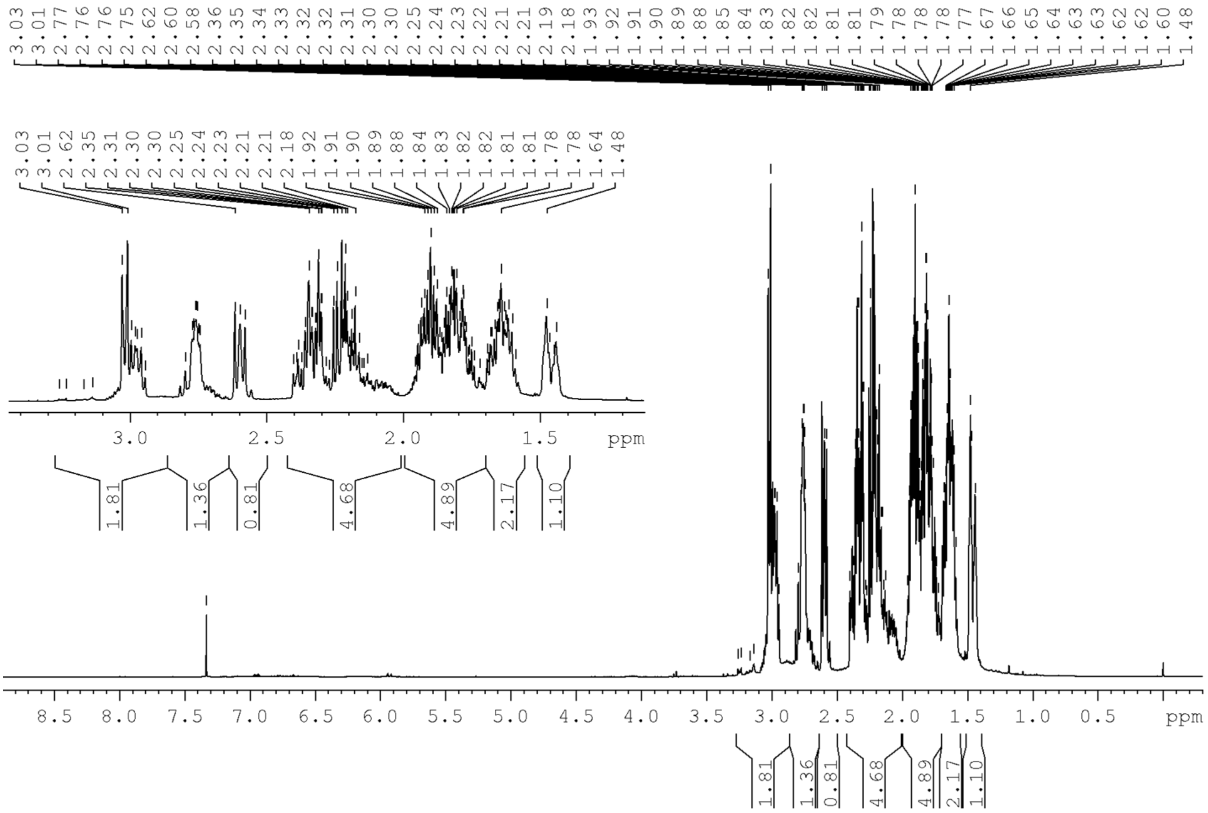

Figure S 20: ^{1}H NMR spectrum of P1(a-d) (400 MHz, $CDCl_3$).

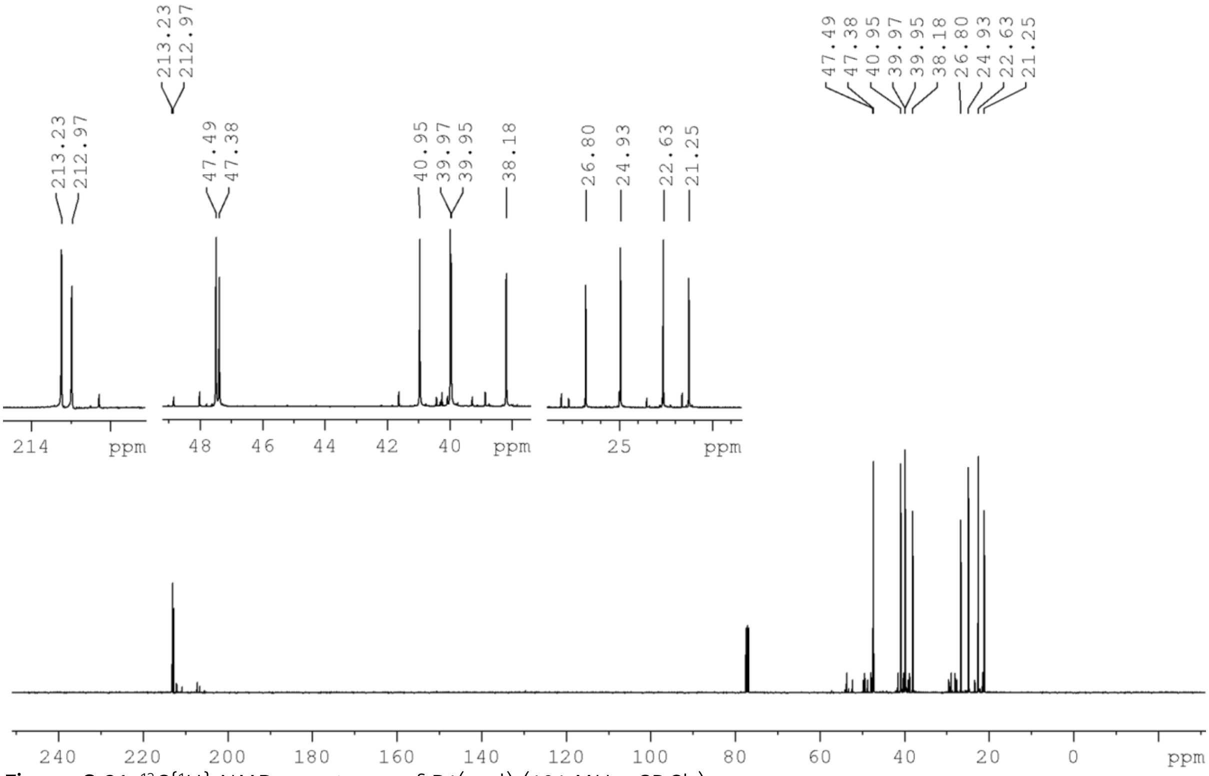

Figure S 21: $^{13}C\{^{1}H\}$ NMR spectrum of P1(a-d) (101 MHz, $CDCl_3$).

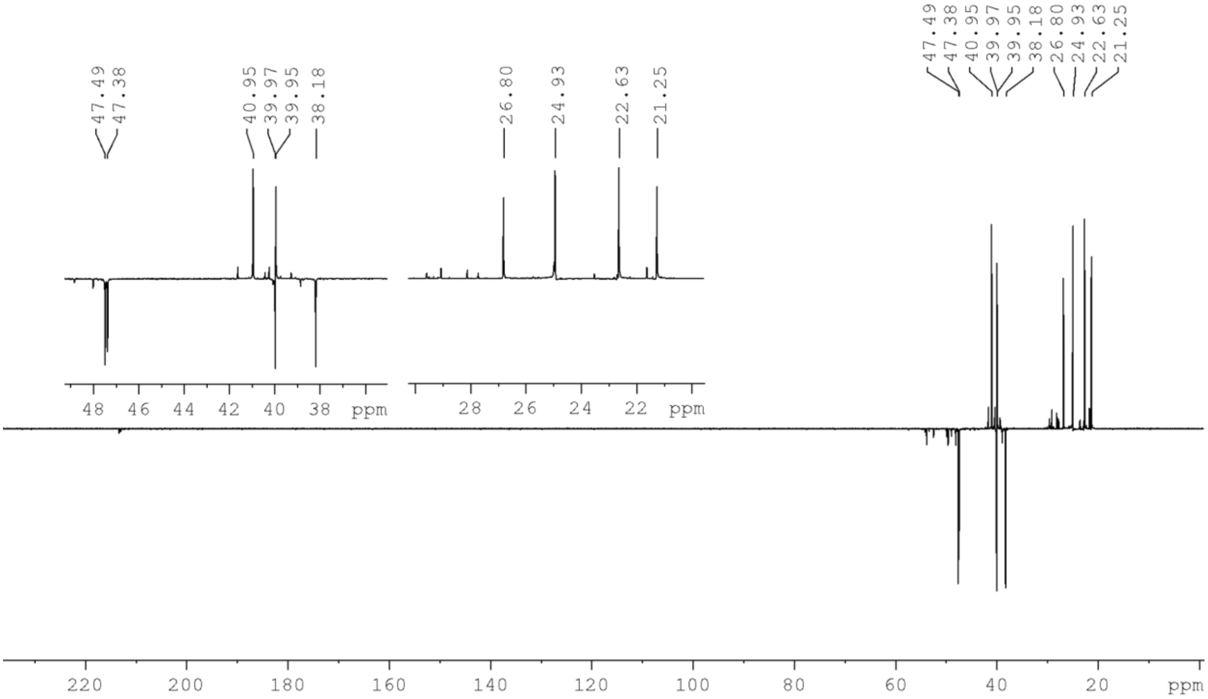

Figure S 22: DEPT-135 $^{13}C\{^1H\}$ NMR spectrum of P1(a-d) (101 MHz, $CDCl_3$).

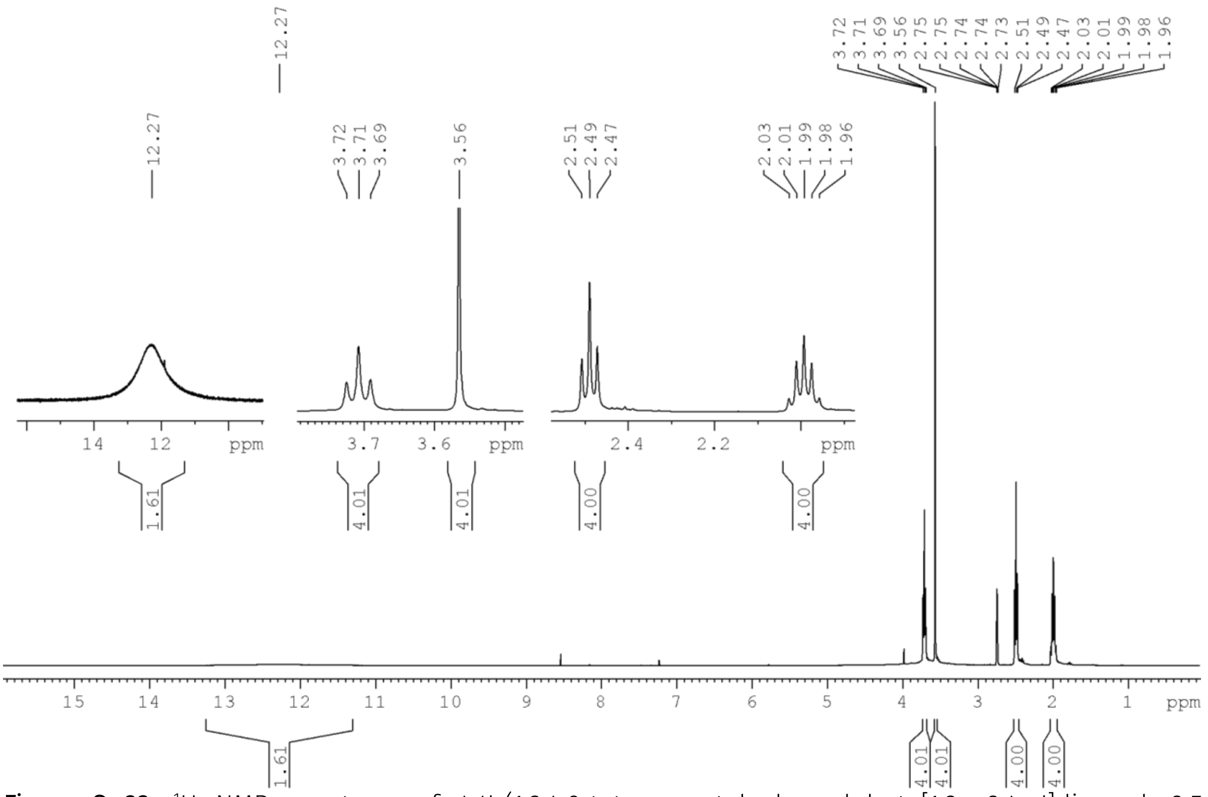

Figure S 23: ^{1}H NMR spectrum of 4,4'-(1,3,4,6-tetraoxooctahydrocyclobuta[1,2-c:3,4-c']dipyrrole-2,5-diyl)dibutyric acid (P2) (400 MHz, $(CD_3)_2SO$).

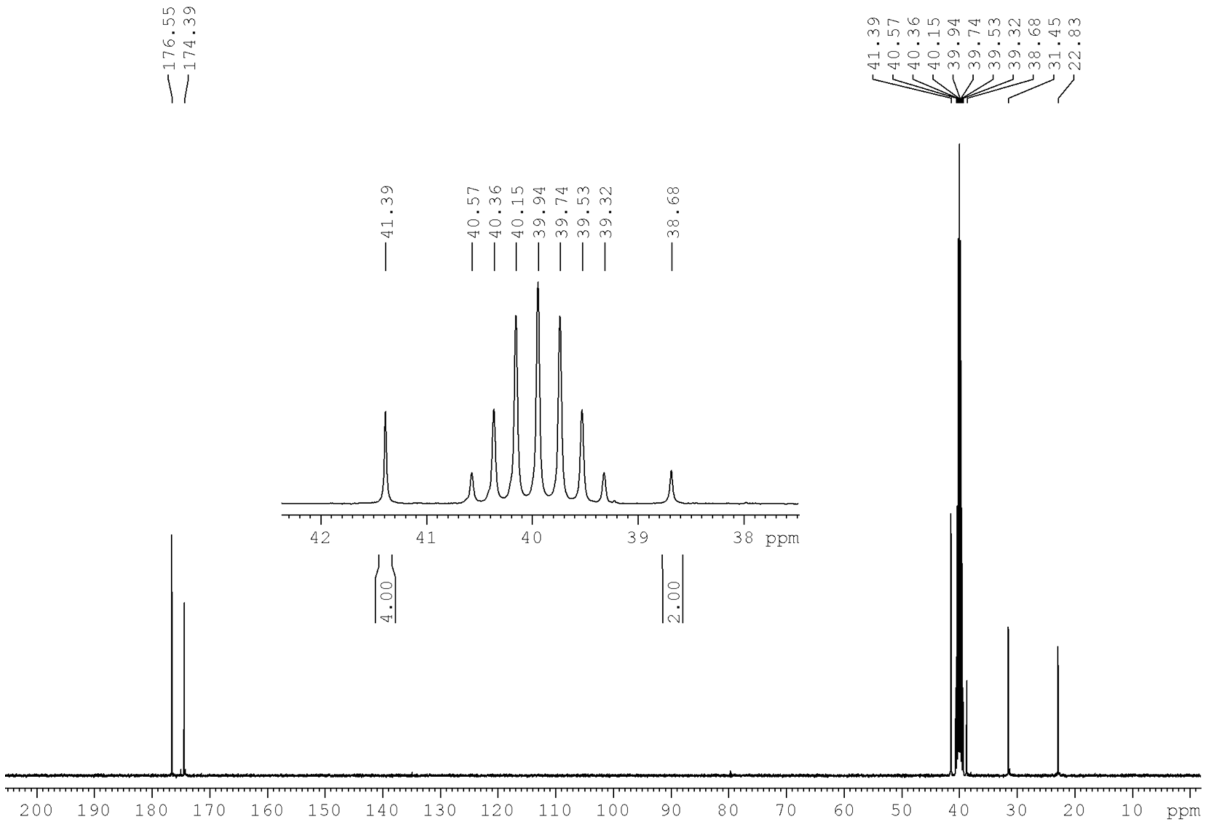

Figure S 24: $^{13}C\{^{1}H\}$ NMR spectrum of 4,4'-(1,3,4,6-tetraoxooctahydrocyclobuta[1,2-c:3,4-c']dipyrrole-2,5-diyl)dibutyric acid (P2) (101 MHz, $(CD_3)_2SO$).

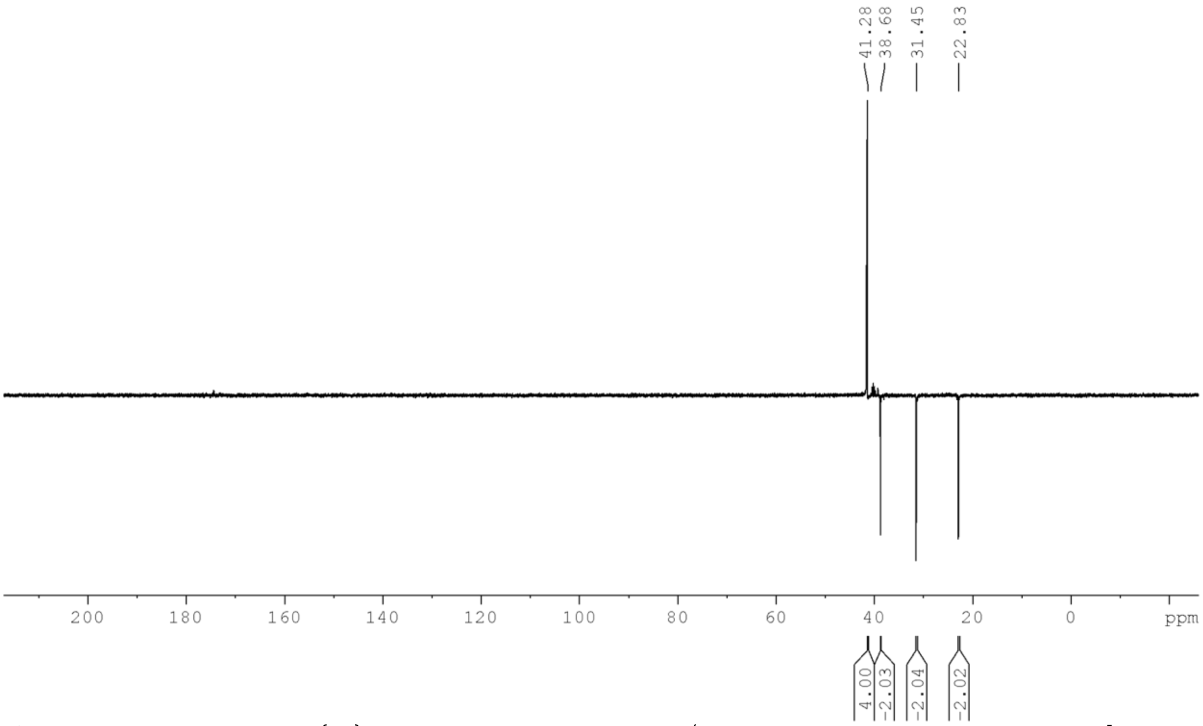

Figure S 25: DEPT-135 $^{13}C\{^{1}H\}$ NMR spectrum of 4,4'-(1,3,4,6-tetraoxooctahydrocyclobuta[1,2-c:3,4-c']dipyrrole-2,5-diyl)dibutyric acid (P2) (101 MHz, $(CD_3)_2SO$).

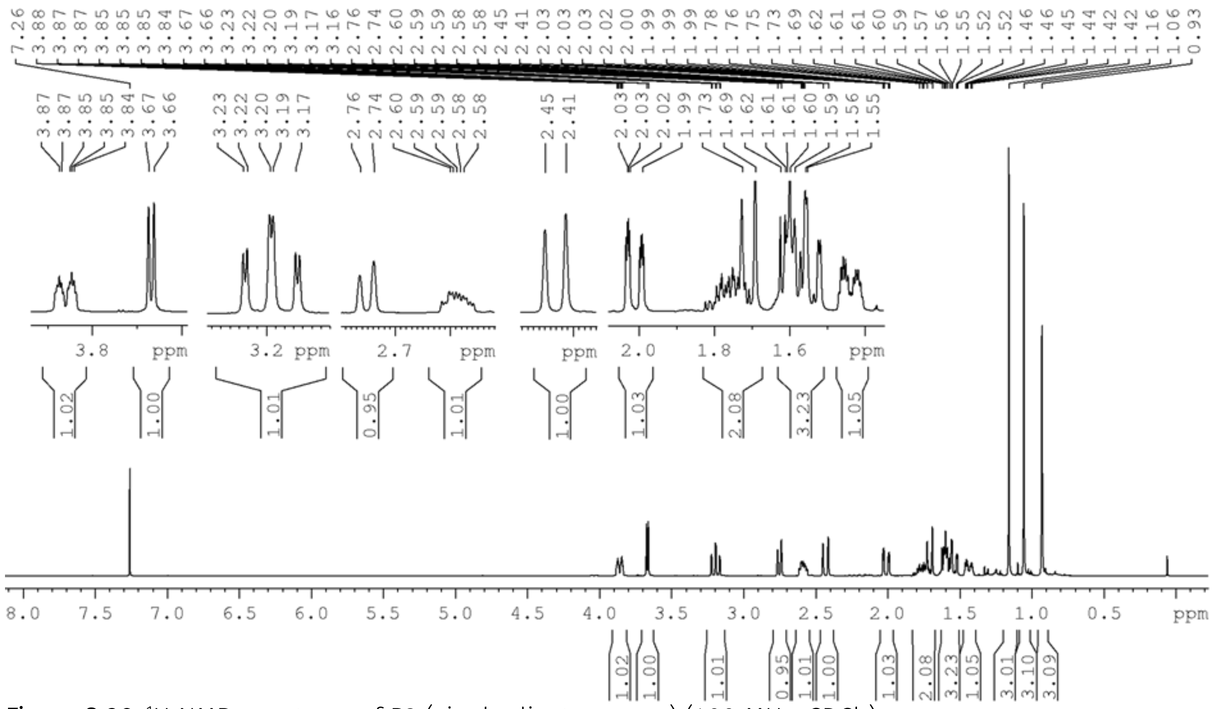

Figure S 26: ^{1}H NMR spectrum of P3 (single diastereomer) (400 MHz, $CDCl_3$).

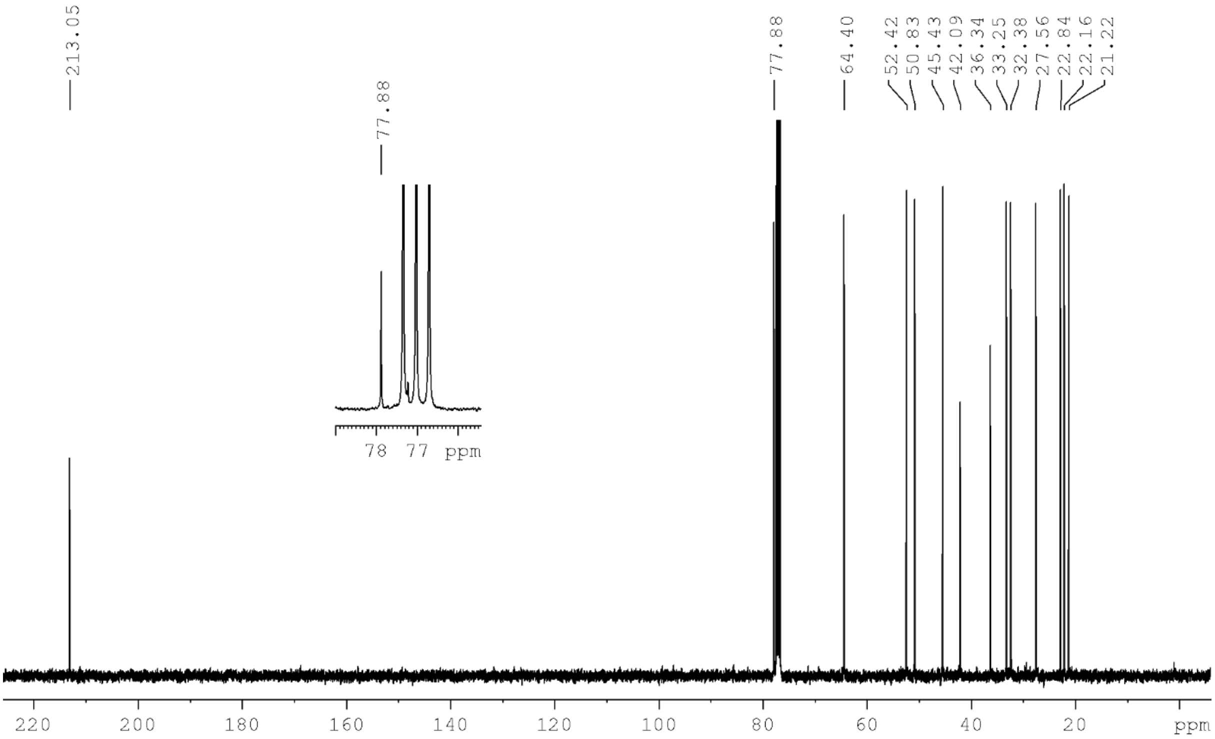

Figure S 27: $^{13}C\{^{1}H\}$ NMR spectrum of P3 (single diastereomer) (101 MHz, $CDCl_3$).

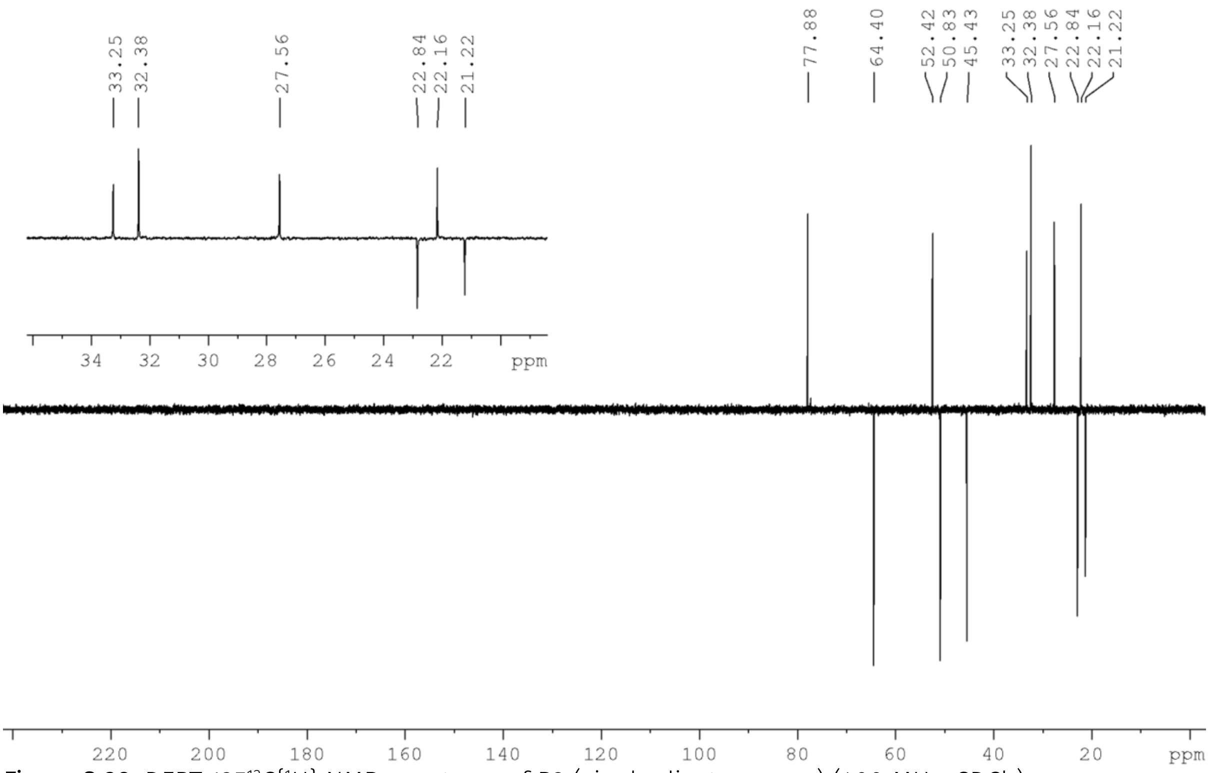

Figure S 28: DEPT-135 $^{13}C\{^{1}H\}$ NMR spectrum of P3 (single diastereomer) (400 MHz, $CDCl_3$).

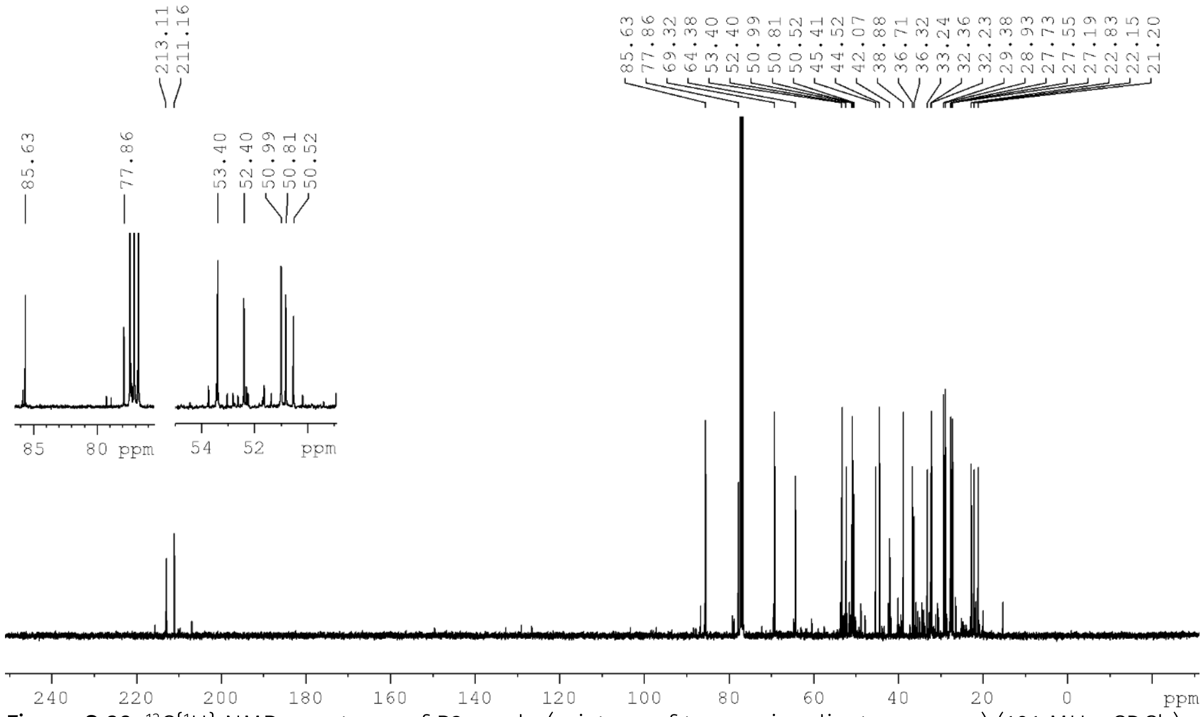

Figure S 29: $^{13}C\{^1H\}$ NMR spectrum of P3 crude (mixture of two major diastereomers) (101 MHz, $CDCl_3$).

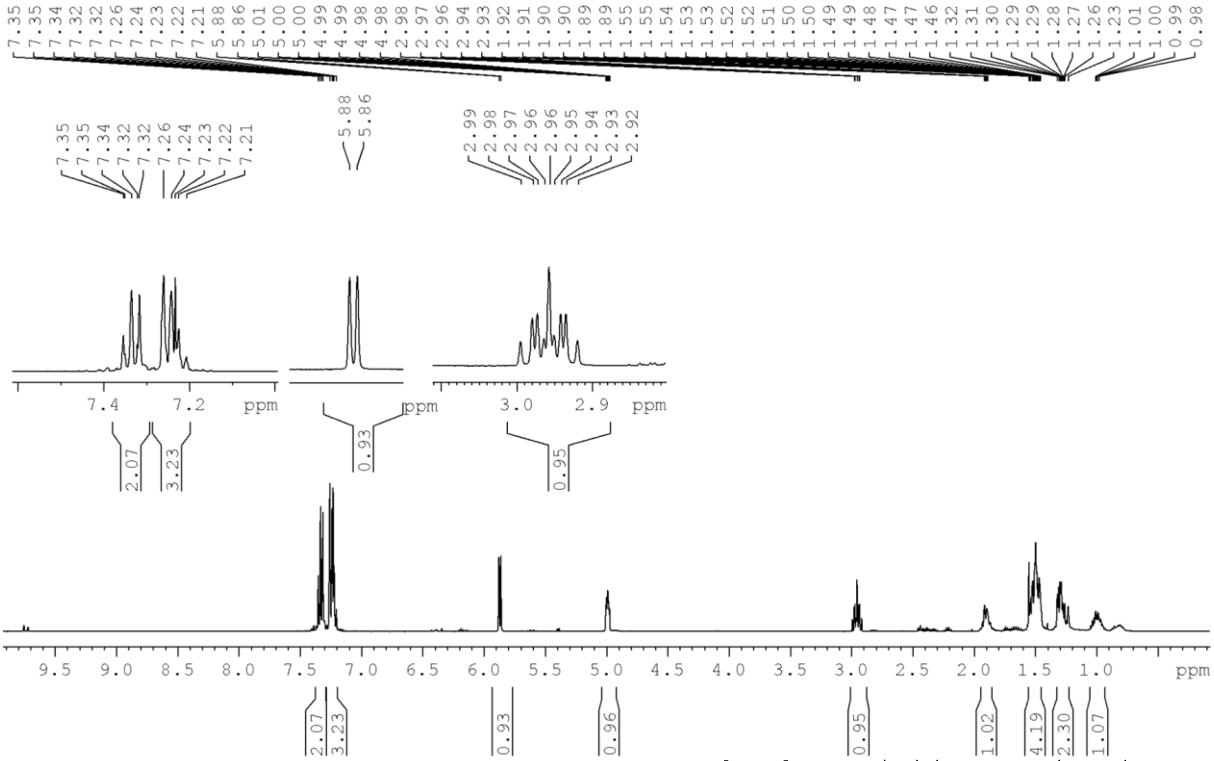

Figure S 30: ^{1}H NMR spectrum of endo-8-phenyl-7-oxabicyclo[4.2.0]octane (P4) (400 MHz, ($CDCl_3$).

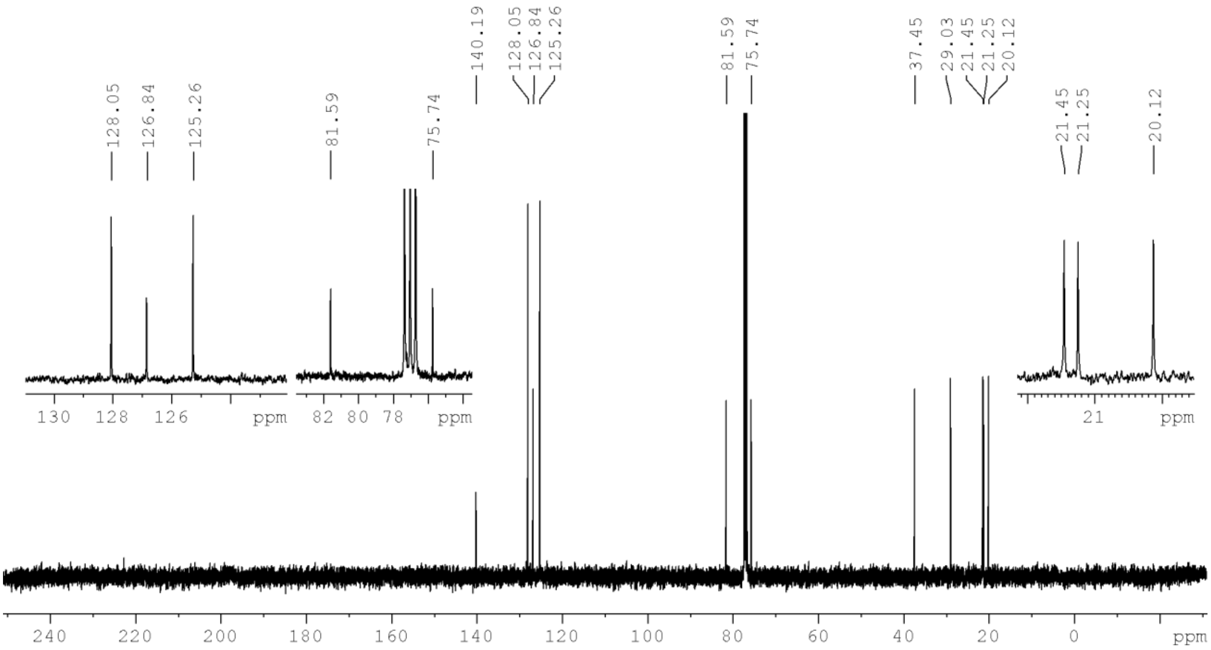

Figure S 31: $^{13}C\{^{1}H\}$ NMR spectrum of endo-8-phenyl-7-oxabicyclo[4.2.0]octane (P4) (400 MHz, ($CDCl_3$)).

References

[1] A. Schroter, S. Märkl, N. Weitzel, T. Hirsch, *Adv. Funct. Mater.* **2022**, *32*, 2113065.

[2] S. Wilhelm, M. Kaiser, C. Würth, J. Heiland, C. Carrillo-Carrion, V. Muhr, O. S. Wolfbeis, W. J. Parak, U. Resch-Genger, T. Hirsch, *Nanoscale* **2015**, *7*, 1403.

[3] S. F. Himmelstoß, T. Hirsch, *Part & Part Syst Charact* **2019**, *36*.

[4] J. Yang, M. B. Dewal, S. Profeta, M. D. Smith, Y. Li, L. S. Shimizu, *J. Am. Chem. Soc.* **2008**, *130*, 612.

[5] J. Yang, M. B. Dewal, L. S. Shimizu, *J. Am. Chem. Soc.* **2006**, *128*, 8122.

[6] K. Kōshi, K. Hiroshi, K. Takahito, CN103571502B, **2014**.

[7] A. G. Griesbeck, S. Stadtmueller, *J. Am. Chem. Soc.* **1991**, *113*, 6923.

[8] N. Harada, Y. Sasaki, M. Hosoyamada, N. Kimizuka, N. Yanai, *Angew. Chem. Int. Ed.* **2021**, *60*, 142.

[9] T. J. B. Zähringer, J. A. Moghtader, M.-S. Bertrams, B. Roy, M. Uji, N. Yanai, C. Kerzig, *Angew. Chem. Int. Ed.* **2023**, *62*, e202215340.

[10] J. Vuilleumier, G. Gaulier, R. de Matos, D. Ortiz, L. Menin, G. Campargue, C. Mas, S. Constant, R. Le Dantec, Y. Mugnier et al., *ACS Appl. Mater. Interfaces* **2019**, *11*, 27443.
